# Upper Limb Motor Function Rehabilitation System Leveraging Pressure‐Sensitive Intent Recognition

**DOI:** 10.1002/advs.202505259

**Published:** 2025-07-11

**Authors:** Zhiwei Hu, Yongchao Yin, Xiaoli Yang, Hanyang Zhang, Ling Xing, Shiwu Zhang, Xinglong Gong, Ming Wu, Guolin Yun, Shuaishuai Sun

**Affiliations:** ^1^ The First Affiliated Hospital of USTC, CAS Key Laboratory of Mechanical Behavior and Design of Materials, Institute of Humanoid Robots, Department of Precision Machinery and Precision Instrumentation University of Science and Technology of China Hefei Anhui 230026 China; ^2^ CAS Key Laboratory of Mechanical Behavior and Design of Materials, Department of Modern Mechanics University of Science and Technology of China Hefei Anhui 230026 China; ^3^ The First Affiliated Hospital of USTC, Division of Life Sciences and Medicine University of Science and Technology of China Hefei Anhui 230001 China

**Keywords:** force‐sensitive composite, functional electrical stimulation, intention recognition, motor function reconstruction system, post‐stroke patient, rehabilitation

## Abstract

Functional electrical stimulation (FES) enhances daily living and rehabilitation outcomes for stroke patients but faces challenges in intention recognition, individual adaptability, and closed‐loop control. To address these, a reconstruction system is presented for upper limb motor function, employing highly pressure‐sensitive liquid metal magnetorheological elastomers (LMMRE） to detect muscle surface pressure. It can dynamically modulate functional electrical stimulation based on muscle force signals, enabling continuous execution of wrist movements for stroke patients. The system markedly improves patient mobility, with wrist lifting angles increased from 14° to 47°, and lifting speed nearly doubled. Clinical trials confirm the system's superior efficacy in promoting motor recovery and reducing muscle spasms. Moreover, functional near‐infrared spectroscopy highlights enhanced brain activity and promotes reconstruction of damaged neural pathways during LMMRE‐FES therapy compared with the conventional electrical stimulation. This system holds immense potential for advancing stroke rehabilitation, paving the way for portable, wearable devices optimized for home‐based care.

## Graphical abstract

A novel upper limb rehabilitation system for stroke patients combines liquid metal elastomer sensors with functional electrical stimulation (FES). By automatically adjusting stimulation intensity based on real‐time muscle force signals, the system enhances wrist movement capability (14° to 47° elevation) and aids neural recovery through heightened brain activity during rehabilitation.

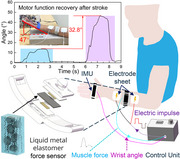

## 1 Introduction

Stroke is a leading cause of mortality and disability worldwide, leaving many survivors with upper limb motor impairments that demand intensive and prolonged rehabilitation.^[^ ^1^, ^2^, ^3^, ^4^, ^5^ ^]^ Effective recovery necessitates high‐intensity, repetitive training to optimize voluntary motor function and minimize compensatory movements.^[^ ^6^ ^]^ To support such efforts, numerous rehabilitation robots,^[^ ^7^, ^8^, ^9^, ^10^, ^11^, ^12^, ^13^ ^]^ including exoskeleton systems, have been developed. Compared to traditional manual physical therapy, these robotic solutions offer superior efficacy at reduced costs. However, current upper limb exoskeletons face limitations such as high equipment costs, large spatial requirements, cumbersome wearability, and expensive maintenance, restricting their feasibility in community‐based and home rehabilitation settings.^[^ ^14^, ^15^, ^16^ ^]^ Consequently, wearable rehabilitation technologies emerge as pivotal for the large‐scale deployment of rehabilitation devices.

Significant progress has been made in wearable assistive devices for post‐stroke rehabilitation, with Functional Electrical Stimulation (FES) based on intent recognition standing out as a key innovation.^[^ ^17^, ^18^, ^19^, ^20^, ^21^, ^22^ ^]^ These systems integrate two core components: an intent recognition module and an FES module. Intent recognition relies on specialized sensors to capture physiological signals and analyze movement intentions. Advanced technologies, including Electroencephalography (EEG),^[^ ^8^, ^24^ ^]^ Electromyography (EMG),^[^ ^19^, ^20^, ^23^, ^25^, ^26^ ^]^ and Force Myography (FMG),^[^ ^27^, ^28^, ^29^ ^]^ enable the recognition of complex movement intentions. FES facilitates motor function restoration by delivering low‐frequency electrical pulses^[^ ^30^ ^]^ to stimulate muscle contractions.

However, stroke‐induced motor impairments weaken limb control and diminish signal strength, complicating intent recognition. EEG, though highly informative, is susceptible to motion artifacts, autonomic nervous activity, and environmental noise, resulting in low signal‐to‐noise ratios and challenging its practicality in daily applications.^[^ ^31^, ^32^, ^33^, ^34^ ^]^ Surface EMG (sEMG) signals are similarly affected by sweating, electrode displacement, and muscle fatigue, reducing system robustness over time.^[^ ^35^, ^36^, ^37^ ^]^ In contrast, FMG technology offers higher stability and reliability due to its non‐invasive nature and superior signal‐to‐noise ratio. By detecting muscle‐tendon stiffness changes using pressure sensors, FMG achieves higher accuracy in intent recognition.^[^ ^38^, ^39^, ^40^, ^41^ ^]^ Despite this, traditional active FES systems often rely on threshold‐based or proportional control mechanisms,^[^ ^42^, ^43^, ^44^ ^]^ lacking individualized real‐time adjustments to stimulation intensity, which can lead to muscle spasms and fatigue, thereby limiting their clinical efficacy.

To address these challenges, this study introduces an upper limb motor function reconstruction system that integrates real‐time pressure detection with intent recognition algorithms for dynamic FES modulation. At its core is a liquid metal magnetorheological elastomer (LMMRE) with anisotropic piezoresistive properties, exceptional sensitivity, and flexibility.^[^ ^45^, ^46^, ^47^, ^48^ ^]^ This material enhances the precision and stability of FMG‐based intent recognition. Furthermore, the dynamic electrical stimulus modulation algorithm possesses adaptive capabilities to accommodate individual differences, overcoming limitations in existing threshold‐based intention recognition. Additionally, we incorporate inertial measurement units (IMUs) for precise wrist motion analysis and motor performance evaluation. Clinical trials demonstrate significant improvements in wrist mobility, motor control, and muscle spasm reduction, validated through several clinical evaluation tools (Figure S1, Supporting Information), including the Fugl‐Meyer Assessment, Brunnstrom Staging, ADL score and the Ashworth Scale. Furthermore, functional near‐infrared spectroscopy (fNIRS) to assess functional connectivity within brain regions during rehabilitation. The results proves that our LMMRE‐FES technology is capable of stimulating brain activity and repairing damaged neural connections more effectively compared to passive functional electrical stimulation. Our system dynamically adjusts electrical stimulation parameters based on intention recognition, resulting in superior rehabilitation outcomes. Our approach offers a transformative solution for stroke rehabilitation, enabling effective, portable, and user‐friendly home care.

## 2 Results

### 2.1 The LMMRE‐FES Integrated System

**Figure 1:**
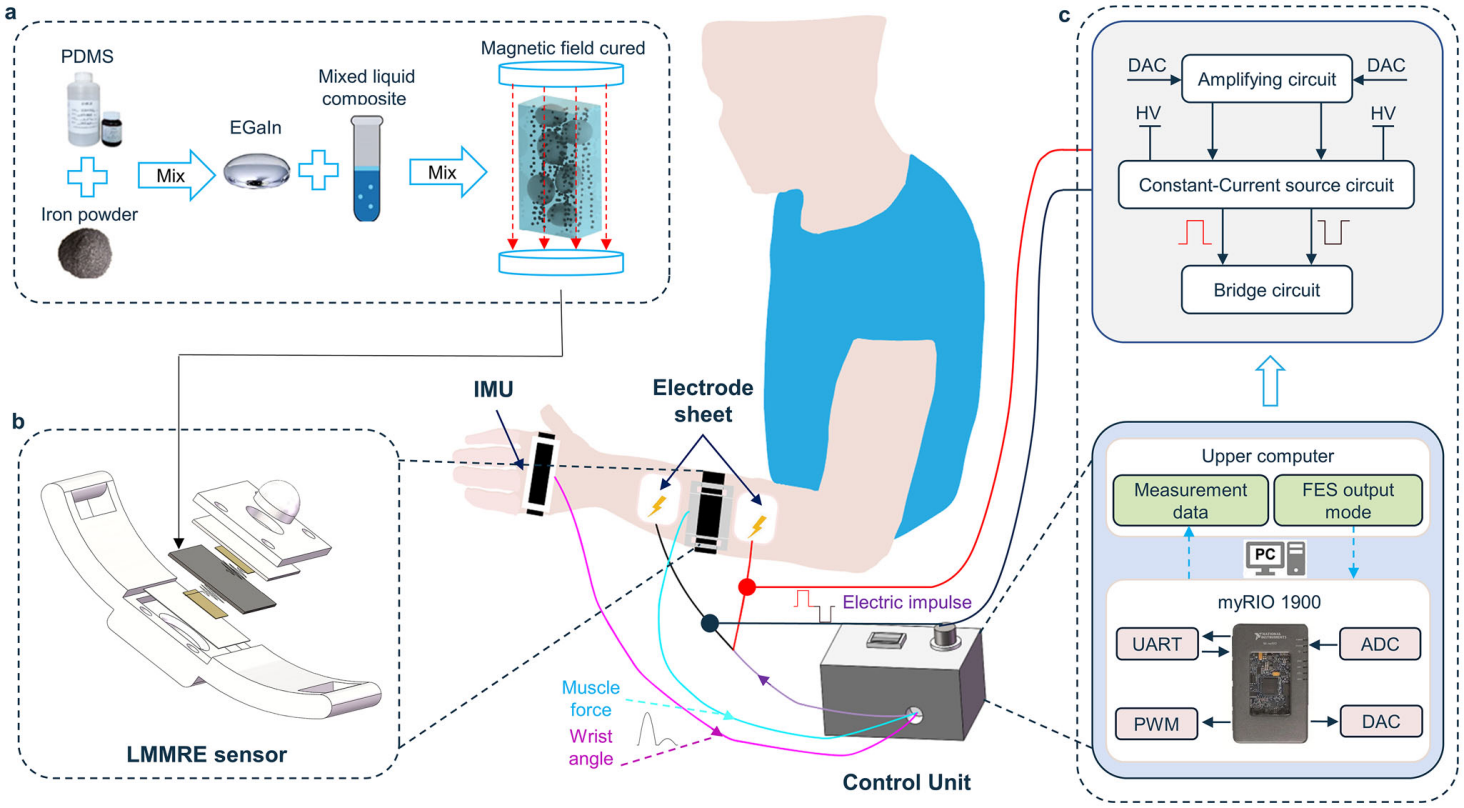
The schematic of the proposed LMMRE‐FES integrated system. a) Production of the LMMRE. b) Sensor structure diagram. c) Detail schematic of the system.

The LMMRE‐FES system consists of an LMMRE sensor, an electrical stimulation module, a controller unit, and an Inertial Measurement Unit (IMU) module (**Figure** **1**; Figure S2, Supporting Information). LMMRE is prepared using polydimethylsiloxane (PDMS) matrix embedded with carbonyl iron particles and liquid metal microdroplets (Figure 1a). The LMMRE sensor detects changes in muscle stiffness and shape through FMG signals to modulate electrical stimulation (Figure 1b); The electrical stimulation module is responsible for generating tunable biphasic square wave pulses for patient treatment.

When a person tries to exercise, nerve signals from the brain's primary motor cortex prompt target muscle contractions (Figure 1c). These changes are recorded by the wearable LMMRE sensor and transmitted to the host computer via the MyRio1900 controller for processing and analysis, which subsequently controls the electrical stimulation circuit output pulses to promote the extension of wrist and fingers. Among them, the LMMRE sensor's high sensitivity to subtle muscle surface pressure changes in stroke patients significantly improve the FMG signal acquisition quality, enabling real‐time accurate judgment of motion intentions. In addition, IMU captures the dynamic behavior of wrist joint, including angle and angular velocity, facilitating the evaluation of exercise rehabilitation progress. To minimize patient discomfort and fatigue, the electrical stimulation module delivers biphasic square‐wave pulse, providing a more comfortable treatment experience. In conclusion, our LMMRE‐FES system integrates the high‐precision intention recognition with FES, offering an effective solution for improving the sports rehabilitation outcomes.

### 2.2 The LMMRE Sensor

Traditional sandwich piezoresistive structures usually consist of three components: the top electrode, the sensing layer, and the bottom electrode. Under applied pressure, the elastic medium compresses, resulting in a reduction in resistance. However, conventional piezoresistive sensing materials often suffer from non‐linear resistance changes with increasing pressure during compression,^[^ ^49^ ^]^ leading to compression saturation and limiting pressure sensitivity. In addition, when the sensor detects muscle group changes on the limb surface, the raised contact part resulting from the narrow displacement during compression may cause discomfort to the user.

**Figure 2:**
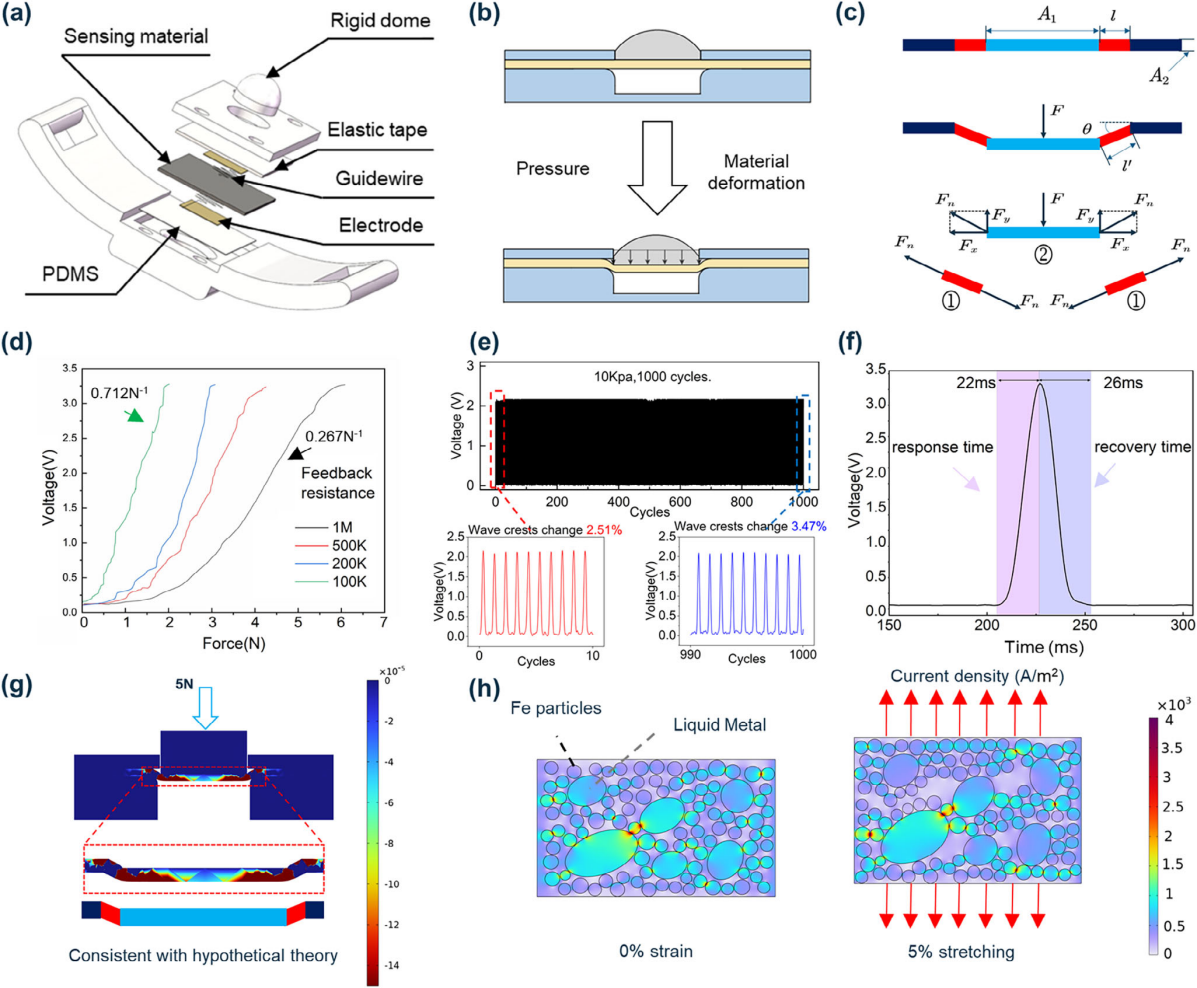
Sensor schematic diagram and simulation. a) Sensor structure diagram.b) Schematic diagram of the sensing structure. c) Schematic diagram of the force applied to the sensing material. d) Output response of the sensor with different feedback resistances. e) Characterization of force repeatability of sensors. f) Sensor response at low loads. g) Strain simulation of materials in this structure. h) Current density simulation of sensing material. (Potential distribution of the material before and after deformation).

To address these issues, we propose a flexible sensing structure based on a pressurized sphere‐LMMRE‐PDMS design (**Figure** **2**a). The substrate layer of the LMMRE sensor is composed of a PDMS thin film, with electrodes positioned on both sides of the LMMRE base layer. The elastic double‐sided tape serves as an isolation layer, securing the rigid pressure sphere and sensing material while providing the sensor with elasticity under external loads. To improve contact stability and prevent electrode detachment from direct skin contact, a rigid spherical cap is used to provide favorable force transfer to the LMMRE. When the sensor is applied to the skin, the cap slightly sinks yet remains firmly attached to the skin, ensuring convenient and reliable transmission of muscle force. Note that using a rigid sphere will produce significant stress concentration on the LMMRE, enhancing sensor sensitivity but increasing the risk of material damage and electrode detachment. Therefore, we choose a rigid spherical cap to balance sensitivity and durability. This structure transforms the compressive strain‐dominated “hard deformation” of the sensing material into a tensile strain‐dominated “soft deformation” (Figure 2b), effectively enhancing sensitivity and user comfort. This design leverages the flexibility of the material to enhance detection of weak pressure on the muscle surface during movement. Unlike traditional structures that only withstand longitudinal pressure, our new design accommodates both longitudinal pressure and transverse tension. This allows greater deformation under the same load, producing more substantial changes in material thickness and resistance, leading to a higher sensitivity. The increased material deformation also enables a larger displacement range for the pressurized ball, ensuring softer skin contact and improved wearing comfort.

Based on the conductive mechanism of the material (Text S1, Supporting Information), we can derive the relationship between resistance and strain as follows:

(1) $$R=R_{0}\left(1-\varepsilon_{y}\right)e^{-p\varepsilon_{y}}$$

where *R*_0_ is the initial resistance of the material, ε_*y*_ is the longitudinal strain of the material, and 𝑝 is a relevant constant term (related to the force area, material properties, etc.).

From the aforementioned equation, it is evident that the sensor's sensitivity, as defined by the following equation, increases with greater impedance change under the same external loading conditions.

(2) $$s\;=\frac{1}{V_{0}}\frac{dV}{dF_{E}}$$

where 𝑉 and *F_E_* represent the sensor signal and external load, respectively, and *V*_0_ is the signal value at the initial moment.

To facilitate computational efficiency, we employed a homogenization approximation approach for the sensing structure, treating the load‐bearing sensing material component as a uniform continuum. Subsequently, we conducted mechanical stress‐strain analysis to evaluate the longitudinal strain response generated by both structural configurations under identical external loading conditions. To ensure valid comparative assessment, we maintained consistent external force magnitudes and contact surface areas across both structural variants. This methodological simplification enabled quantitative comparison of the mechanical transduction efficiency between the conventional and proposed architectures while preserving analytical tractability.

In conventional sandwich configurations, the sensing material undergoes compressive deformation resulting from the combined influence of external loading and interactions with the underlying substrate. Consequently, the elastic modulus of the bottom substrate(*E*_0_) contributes significantly to the material's deformation characteristics (deformation schematics are illustrated in Figure S3, Supporting Information). When compared with the compressive modulus of the sensing material itself (*E*_1_), *E*_0_ ≫ *E*_1_. Therefore, the strain manifested within this structural configuration can be mathematically formulated as:

(3) $$\varepsilon_{0}=\frac{F}{E_{1}A_{1}}$$

where ε_0_ is the longitudinal strain generated by the material under the action of the traditional sandwich structure, *F* is the external load, and *A*_1_ is the compressive action area.

For the hollow sandwich structure proposed in this study, the base elastic modulus *E*_0_′ at this time changes due to the alteration of this parameter by the cavity, resulting in a change in material deformation and an increase in tensile deformation. For analytical convenience, we divided the material deformation into two distinct regions (as shown in Figure 2C). In Region ①, the material undergoes predominantly tensile‐induced deformation, generating a tensile strain characterized by ε_1_. Conversely, in Region ②, the material experiences simultaneous tensile and compressive forces, resulting in a composite longitudinal strain of ε_2_. Consequently, the strain manifestations within these two regions of the hollow structure can be mathematically represented as follows (complete derivation procedures are detailed in Text S2, Supporting Information):

(4) $$\varepsilon_{1}=\frac{1}{2}\;{\left(\frac{F}{E_{2}A_{2}}\right)}^{\frac{2}{3}}$$

(5) $$\varepsilon_{2}=\nu \frac{1}{\sqrt{{\left(\frac{E_{1}A_{1}}{F}\right)}^{\frac{2}{3}}+4{\left(\frac{E_{1}A_{1}}{F}\right)}^{\frac{4}{3}}}}+\frac{F}{E_{2}A_{2}}$$

where *E*_2_ is the tensile modulus of the material, and *A*_2_ is the tensile action area, ν is the Poisson's ratio of the material.

These results demonstrate that the cavity structure exhibits greater deformation compared to traditional sandwich structures.

Due to the concentrated force distribution rather than uniform loading in Region ②, the material exhibits pronounced deformation gradients, with maximal displacement at the peripheral boundaries and minimal displacement in the central domain—a pattern that demonstrates excellent concordance with our finite element simulation results. Despite the larger magnitude of deformation generated through tensile mechanisms, practical implementation constraints, including the limited spatial dimensions and complications associated with electrode lead placement, necessitated positioning the sensor's functional zone at the terminal sections of Region ②. Under this optimized configuration, the longitudinal strain within the operational domain of the material (ε) satisfies the following relationship:

(6) $$\varepsilon_{0}<\varepsilon_{2}<\varepsilon <\varepsilon_{1}$$

The results indicate that our structure increases the longitudinal strain of the material, thereby enhancing the sensor's sensitivity. To further elucidate the sensing mechanism, we analyze the forces acting on the material (Figure 2c; Text S2, Supporting Information).

To characterize the performance of the sensor, a series of tests were conducted. The sensor's sensitivity is significantly influenced by the feedback resistance, as it impacts the gain of the operational amplifier circuit. Real‐time adjustment of the feedback resistance allows for effective control over both sensitivity and sensing range. The response curves of the sensor's signal to external load for different feedback resistances are shown in Figure 2d. As the feedback resistance increases, sensitivity decreases exponentially, while the sensing range expands. Specifically, increasing the feedback resistance from 100 kΩ to 1 MΩ reduced sensitivity from 0.712 N^−1^ to 0.267 N^−1^, but expanded the pressure range by 301%. This trade‐off enables the sensor to extend its pressure range at the cost of some sensitivity, with a simple and fast switching mechanism. Given individual variability, different patients exhibit varying muscle surface pressures during limb movement and require distinct force ranges. This variability can be addressed by adjusting the feedback resistance, allowing for selection of an appropriate measurement range.

A 1000‐cycle cyclic loading test demonstrates the sensor's excellent stability (Figure 2e), with the output voltage difference remaining below 5%, ensuring reliable performance for long‐term monitoring tasks. The sensor's repeatability and consistency are well‐suited for extended use and ease of application. Furthermore, the sensor's response under low load conditions was tested. Under a 0.05 N load, the sensor exhibits a response time of ≈22 ms and a recovery time of ≈26 ms. Such a low detection limit confirms its suitability for motion detection tasks (Figure 2f).

To verify the hypothesis that this structure generates greater strain, we performed finite element simulations of the sensor and piezoresistive composite (See details in Text S3 and Figures S4 and S5, Supporting Information). The strain field analysis for this structure is shown in Figure 2g. Cross‐sectional results under a 5 N load reveal that this design induces greater longitudinal strain and displacement compared to traditional sandwich structures. Additionally, Figure 2h shows the simulated current density distribution across the square surface. As indicated by the color gradient from blue to red, current density increases exponentially with strain. Notably, the current density at the material boundary and in the tensile direction rises significantly at 5% strain. Under tensile strain, the material exhibits a positive Poisson's ratio effect, resulting in longitudinal compression when subjected to transverse stretching. This causes a significant reduction in inter‐particle distances at contact interfaces, leading to decreased contact resistance and consequently increased current density. When inter‐particle gaps are reduced to nanometer scale, quantum tunneling effect^[^ ^50^, ^51^ ^]^ become dominant, allowing electrons to traverse potential barriers between conductive particles even without direct physical contact. This mechanism^[^ ^47^ ^]^ is particularly pronounced at the boundaries of liquid metal microdroplets, where the electrical field concentration effect is most significant. These findings demonstrate that our hollow sandwich sensor structure produces greater strain under the same load than traditional designs, leading to more significant impedance changes and higher sensitivity.

Beyond demonstrating these advantages through simulation, we conducted comparative studies with commercial piezoresistive sensor (IMS‐C10A).The LMMRE sensor showed significantly lower lateral force sensitivity (Figure S18a, Supporting Information) and reduced temperature sensitivity (Figure S18b, Supporting Information) while maintaining comparable sweat sensitivity (Figure S18c, Supporting Information). Extended reliability testing revealed exceptional stability with <5% output variation over 1000 cycles (>1 h), compared to ≈20% drift in commercial sensors. Dynamic skin‐interface testing showed LMMRE maintained 4.3% impedance variation versus 8.7% for piezoresistive sensors across 100 wrist extension cycles (Figure S17, Supporting Information). These results confirm LMMRE's advantages in cross‐sensitivity rejection, long‐term stability, and reliable skin contact for muscle force measurement applications (see details in Text S8, Supporting Information).

### 2.3 Intent Recognition Algorithms and Motor Function Reconstruction Strategies

**Figure 3:**
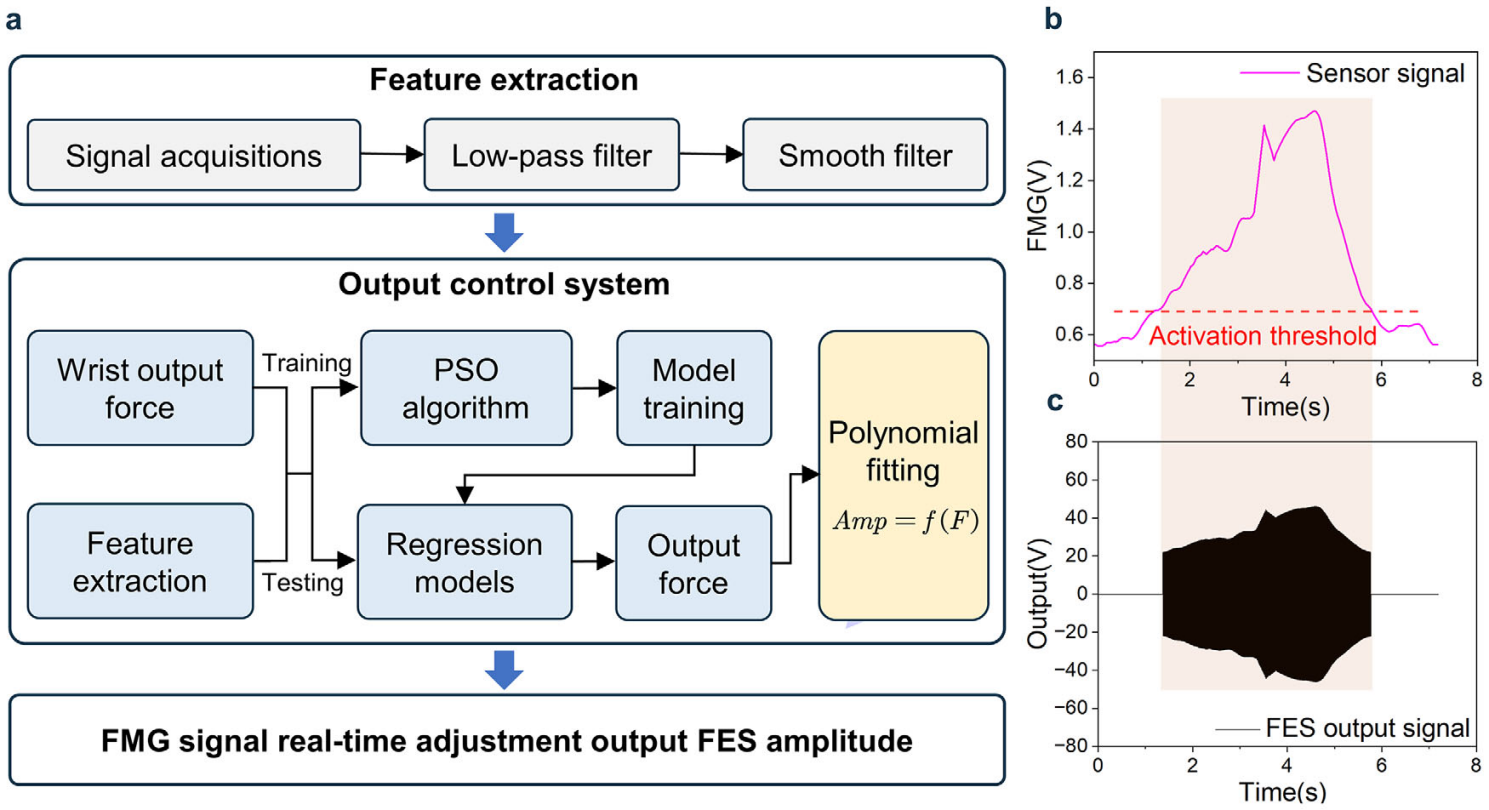
Intent recognition algorithms. a) Algorithm block diagram. b) Surface muscle strength signal for PWM parameter modulation. c) FES output modulation by FMG signals.

In this study, we propose an intention recognition algorithm that integrates least‐squares support vector machine (LS‐SVM) with polynomial fitting (PF) to correlate muscle surface pressure signals with muscle force and FES. SVM, based on statistical learning theory, efficiently addresses challenges associated with small sample sizes, nonlinearity, and high‐dimensional data, offering advantages over neural networks. LS‐SVM, a variant of SVM, simplifies the solution of quadratic programming problems, enabling accurate approximation of nonlinear systems. PF facilitates modeling of the nonlinear relationship between FES‐induced muscle forces and parameters, requiring minimal experimental data and accommodating individual variability. Given the limited training data available in clinical FES applications and the need for smooth output, we combined LS‐SVM and PF to establish the FMG‐FES relationship (Text S4, Supporting Information). The logic block diagram of the algorithm is shown in **Figure** **3**a. The core of the algorithm is to predict the wrist output force based on the processed FMG signal through LS‐SVM regression analysis, using the PSO algorithm to optimize parameters to improve the training speed of the model. At the same time, polynomial fitting is used to map the force to the amplitude, achieving real‐time adjustability of the FES output amplitude during system operation.

The system's control algorithm is implemented in LabVIEW software, running on the host computer (Figure S6, Supporting Information) and MyRio controller. It includes modules for data acquisition, processing, and output control. After collecting the FMG signals, the data is filtered and smoothed. The controller then modulates the pulse width and frequency of the electrical stimulation, delivering variable electrical pulses to the patient's limb via electrodes. During operation, when FMG exceeds a predetermined threshold (usually 30% above the resting state), the FES stimulator will be triggered to generate electrical stimulation, with real‐time amplitude adjustments based on FMG levels (Figure 3b,c). The algorithm allows the system to dynamically adjust electrical stimulation parameters in real time (Figure S7, Supporting Information), effectively accommodating patients' movement needs during training. Compared to traditional electrical stimulation control methods (Figure S8, Supporting Information), this approach enhances control accuracy of FES by incorporating advanced algorithms and models. This enhancement leads to improved movement flexibility and rehabilitation efficiency in subjects, effectively addressing the challenge of individual adaptability.

### 2.4 Upper Limb Rehabilitation Training and Rehabilitation Evaluation

To evaluate the rehabilitation effects on stroke patients, a clinical experiment was conducted (Figure S9, Supporting Information). Participants underwent daily 40‐min sessions of upper limb rehabilitation training, including a 10‐min rest period. The active training group received intent‐recognition‐based electrical stimulation therapy using the LMMRE‐FES system, while the control group received only standard electrical stimulation.

**Figure 4:**
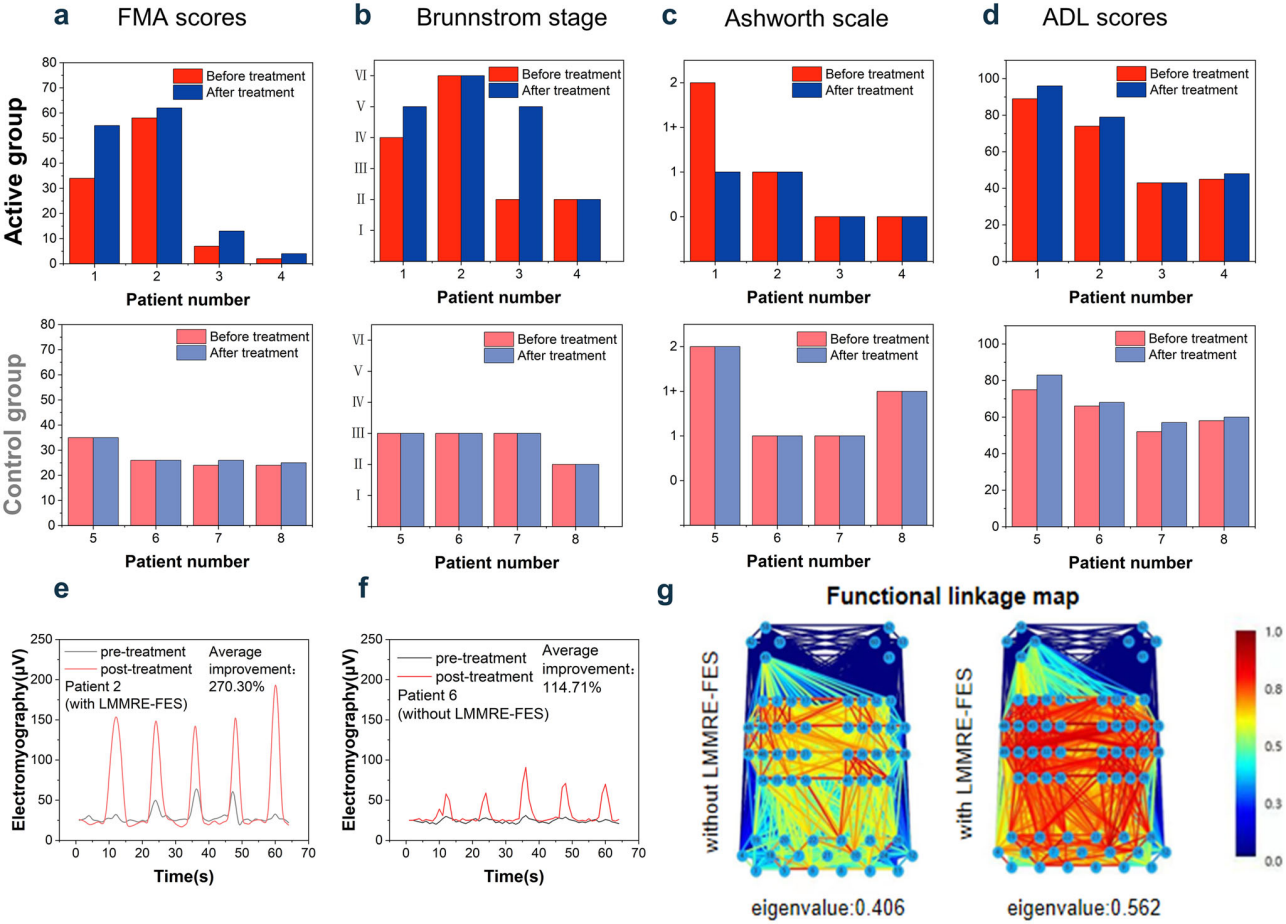
Rehabilitation by LMMRE‐FES. a) Comparison of FMA‐UE scores before and after treatment. b) Comparison of Brunnstrom stages before and after treatment. c) Comparison of Ashworth Scale scores before and after treatment. d) Comparison of ADL scores before and after treatment. e) Comparison of EMG assessment of patient 2(with LMMRE‐FES) before and after treatment. f) Comparison of EMG assessment of patient 6(without LMMRE‐FES) before and after treatment. g) fNIRS data acquisition and analysis.

Before and after the two‐week trial, standardized assessments, including the Fugl‐Meyer Assessment (FMA), Brunnstrom Staging, Modified Ashworth Scale, and Activities of Daily Living (ADL) assessment, were performed to evaluate physical function. As shown in Figure 4a–d, the active training group achieved a 63.59% average improvement in FMA scores compared to 3.13% in the control group, demonstrating significant recovery in extension on the affected side. Brunnstrom stages improved by one level in the active group but remained unchanged in the control group, indicating enhanced upper limb and hand strength. Modified Ashworth Scale scores in the active group decreased by 0.5 points on average, reflecting reduced wrist stiffness and accelerated motor recovery, while no change was observed in the control group (Text S5, Supporting Information). Both groups exhibited a 6% increase in ADL scores, suggesting general improvements in daily living abilities. These findings collectively indicate superior outcomes for patients treated with LMMRE‐FES compared to conventional FES. Additionally, participants underwent follow‐up evaluation 3 months post‐intervention to assess the durability of therapeutic improvements. Results demonstrated sustained therapeutic benefits, with motor function gains maintained at levels comparable to immediate post‐treatment assessments (see details in Text S9 and Table S2, Supporting Information).

EMG assessments further supported these findings. As depicted in Figure 4e,f, two right‐handed subjects with affected right sides were selected for analysis. Subject 2 from the active group exhibited a 270.3% improvement in mean EMG signal strength, significantly outperforming the 114.71% improvement in subject 6 from the control group. These results underscore the faster recovery of upper limb and hand strength with LMMRE‐FES treatment.

Additionally, near‐infrared spectroscopy (NIRS) analysis demonstrated enhanced brain activity during LMMRE‐FES therapy compared with the pure FES treatment. As shown in Figure 4g, subject 1 exhibited a 38.42% increase in mean NIRS functional intensity eigenvalue (0.562) compared to FES alone (0.406 in the control group), indicating heightened brain activity and significant changes in Hemoglobin Oxygenated (HbO) concentration when LMMRE‐FES was combined with intent recognition (Figure S10, Supporting Information). This means the LMMRE‐FES can promote the reconstruction of damaged neural pathways in the brain compared with the conventional FES technology.

### 2.5 Motor Function Reconstruction

**Figure 5:**
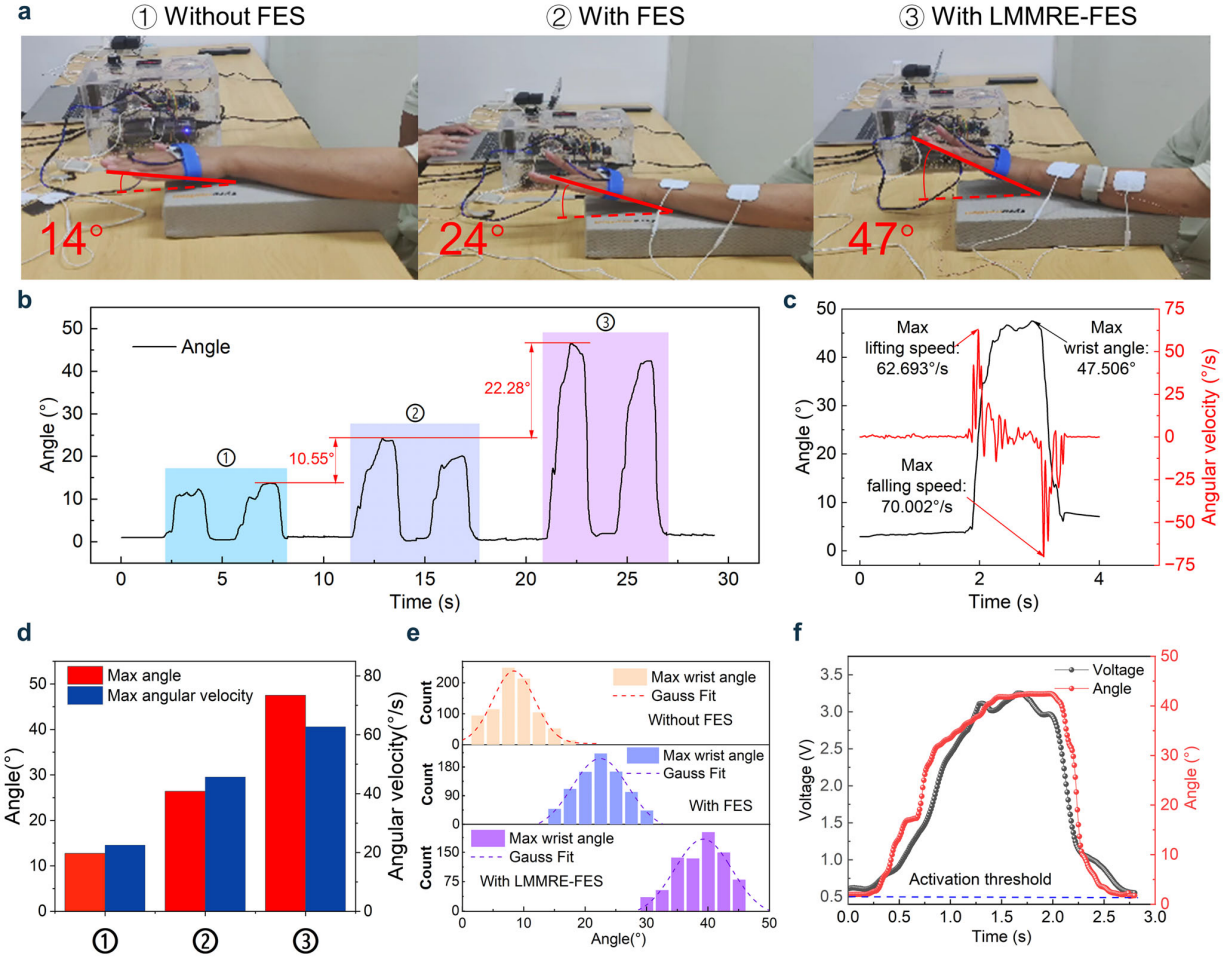
Motor function reconstruction of LMMRE‐FES. a) Comparison of wrist lift angle of patient without wearing device, single electrical stimulation, intention recognition + electrical stimulation. b) Changes in angle of wrist lift in patient with no device, single electrical stimulation, intention recognition + electrical stimulation. c) Changes in angle and angular velocity of wrist lift in patient with intention recognition + electrical stimulation. d) Changes in max angle and max angular velocity of wrist lift in patient with no device, single electrical stimulation, intention recognition + electrical stimulation. e) The maximum angle distribution of patient without wearing device, single electrical stimulation, intention recognition + electrical stimulation. f) FMG and angle signals during wrist lifting.

The results of functional reconstruction in active training group and control group were statistically analyzed. **Figure** **5**a depicts wrist elevation angles under three conditions: no FES, traditional FES, and LMMRE‐FES. Subjects achieved wrist elevations of ≈14° without FES, 24° with traditional FES, and 47° with LMMRE‐FES. Compared to no FES, traditional FES improved wrist elevation by 71.43%, while LMMRE‐FES demonstrated a 235.71% improvement, underscoring its superior efficacy in motor function reconstruction. Detailed angle variations in Figure 5b and Figure S11 (Supporting Information) further highlight the significant advantages of LMMRE‐FES. Figure 5c presents changes in wrist elevation angle and angular velocity with LMMRE‐FES, achieving a maximum angular velocity of 62.693°/s. The changes observed in the other two cases are presented in Figure S12 (Supporting Information). Figure 5d compares maximum wrist angles and velocities, showing a 37.32% improvement in angular velocity with LMMRE‐FES due to its precise regulation of FES, which enhances muscle coordination. Figure 5e visualizes the distribution of maximum wrist elevation angles, further emphasizing LMMRE‐FES's superior motor function enhancement (Figure S13, Supporting Information). Additionally, Figure 5f records FMG and angle signals during wrist elevation (Figure S14, Supporting Information). These results demonstrate that LMMRE‐FES significantly improves motion performance, accuracy, and motor function recovery.

Traditionally, it was believed that muscles contracted immediately after electrical stimulation, making it challenging for subjects using conventional FES systems to control their muscles effectively during experiments. In contrast, the LMMRE‐FES system requires subjects to autonomously initiate wrist movement to activate FES. This approach pre‐activates synergistic muscles associated with the intended motion, enhancing muscle contraction upon electrical stimulation. The combined effect of the subject's voluntary movement and stimulation‐induced contraction significantly improves wrist elevation angle and lifting speed. These results demonstrated that the therapeutic outcomes of the active training group were superior to those of the traditional FES group. Our findings validate the effectiveness and feasibility of FES intervention based on motor intention recognition and voluntary control. This approach not only reconstructs motor function but also enhances the subject's range of motion, offering a promising solution for improving rehabilitation outcomes.

## 3 Discussion

This study presents an efficient Liquid Metal Magnetorheological Elastomer Functional Electrical Stimulation (LMMRE‐FES) system designed to restore upper limb motor function in stroke patients. By utilizing LMMRE sensors to detect muscle surface pressure, the system dynamically adjusts FES output parameters in real‐time, delivering precise electrical stimulation to facilitate wrist and finger movements. This approach not only enhances motor function but also effectively alleviates muscle spasms, as demonstrated in clinical trials.

The integration of LMMRE as a pressure‐sensitive material marks a significant breakthrough in wearable rehabilitation technology. With anisotropic piezoresistive properties and exceptional sensitivity, the LMMRE sensor is capable of detecting pressures as low as 0.05 N, making it highly effective in capturing subtle changes in muscle stiffness and shape. The innovative pressure ball‐LMMRE‐PDMS design further enhances sensitivity by converting compressive strain into tensile strain, improving performance and ensuring user comfort during extended use.

Clinical trials with stroke patients exhibiting varying degrees of motor impairment validated the LMMRE‐FES system's efficacy. Active training using the system yielded remarkable improvements compared to passive training. FMA scores increased by 63.59%, Brunnstrom stages advanced by one level, and Ashworth Scale scores decreased by 0.5 points on average in the active group. EMG data revealed a 270.3% increase in muscle activity, compared to 114.71% in the control group, highlighting significant muscle recovery. fNIRS demonstrated a 38.42% improvement in brain connectivity during LMMRE‐FES stimulation compared with conventional FES, reflecting enhanced neural repairing and coordination. Despite sample heterogeneity in stroke characteristics and baseline motor function, these consistent therapeutic mechanisms suggest that the LMMRE‐FES system exhibits cross‐individual functional adaptability, with therapeutic efficacy characterized by relative motor recovery improvements rather than dependency on absolute baseline functional levels.

Motor function reconstruction experiments further confirmed the system's effectiveness. During arm lifting tasks, LMMRE‐FES intervention improved wrist elevation angle by 80.02% and angular speed by 37.32% compared to traditional FES. By integrating intent recognition, the system activated synergistic muscles associated with patient movement, significantly expanding the range of motion and demonstrating the feasibility of voluntary consciousness‐based FES intervention.

Statistical analysis revealed significant inter‐patient variability (Figure S15, Supporting Information) in wrist elevation performance (F(3,56) = 250.81, p < 0.001, η^2^ = 0.93), with maximum angles ranging from 30.9° to 47.5° across 4 participants. The large effect size underscores the clinical significance of individual differences in stroke rehabilitation outcomes, emphasizing the critical need for personalized therapeutic protocols tailored to each patient's functional capacity and recovery potential. Notably, while extension angles could be increased through intensified electrical stimulation or modified detection thresholds, therapeutic interventions must be tailored to individual patient conditions. Our LMMRE‐FES system achieves wrist extension angles of 40°±5°, which falls within the functional range (20°–45°) established by Kaufman‐Cohen et al. (2018)^[^ ^52^ ^]^ in healthy subjects. Extension angles exceeding this functional range may not yield proportional therapeutic benefits and could increase muscle fatigue or patient discomfort. Therefore, future development will prioritize system stability, user comfort, and personalized parameter calibration to optimize therapeutic outcomes.

Additionally, we conducted comparative analysis between the LMMRE‐FES system and electromyography‐driven electrical stimulation systems. This evaluation confirmed that while both modalities demonstrate synchronized activation patterns and comparable sensitivity for motor intent recognition, the LMMRE‐FES system provides superior mechanical specificity, electromagnetic interference resistance, and long‐term calibration stability, making it particularly advantageous for sustained muscle force monitoring in rehabilitation applications.

In conclusion, the LMMRE‐FES system represents a transformative approach to stroke rehabilitation by combining advanced sensor technology, real‐time intent recognition, and adaptive electrical stimulation. Its ability to provide personalized, dynamic rehabilitation support delivers substantial improvements in mobility and independence for stroke patients.

## 4 Conclusion

This study introduces a functional electrical stimulation (FES) system integrated with LMMRE‐based intention recognition to facilitate autonomous upper limb rehabilitation in stroke patients. The system employs a highly sensitive LMMRE sensor to enhance motor performance by utilizing residual muscle strength signals from the affected limb. This enables intention‐driven control through electrical stimulation, supporting repetitive wrist and finger exercises. Compared with traditional methods, the system significantly enhanced electrical stimulation control through autonomous intent intervention, resulting in a 37.32% increase in the subject's wrist lifting speed and wrist Angle from 24° to 47°. Clinical trials involving eight participants demonstrated notable motor function improvements. Functional near‐infrared spectroscopy further confirmed superior neural coordination and connectivity. This innovative system shows significant promise for stroke rehabilitation, providing a portable, wearable solution for home‐based care.

## 5 Experimental Section

### Preparation of the LMMRE

The raw materials for LMMRE include PDMS (Dow Corning), a two‐component system with vinyl‐terminated polydimethylsiloxane (Part A) and polydimethylsiloxane (Part B); a platinum (Pt) catalyst; carbonyl iron powder (Sigma, 1–5 µm); and liquid metal EGaIn, a Ga‐In alloy with a melting point of 15.5 °C.

The preparation process is shown in Figure 1a. First, PDMS A and B were mixed in a 10:1 mass ratio using an electric mixer at 1000 rpm for 2 min, followed by degassing with a vacuum pump. Iron powder was then added in a 1:2.7 mass ratio (PDMS powder) and manually mixed, followed by electric stirring at 1500 rpm for 5 min to ensure uniform dispersion. The mixture was degassed again until no visible bubbles remain. Subsequently, PDMS, carbonyl iron powder, and liquid metal are combined in a 1:2.7:1 mass ratio, stirred manually until no silver particles were visible, and poured into a pre‐prepared mold. The mold was degassed again, placed in a uniform 1T magnetic field perpendicular to the mold, and cured at 100 °C for 20 min. The final anisotropic LMMRE composite was obtained after natural cooling.

### Fabrication of the LMMRE Sensor

The manufacturing process for the new structural sensors consists of several steps. First, the sensor housing was designed and fabricated using light‐curing resin, with nylon Velcro straps ensuring stable wearability. Electrodes made of conductive copper foil were placed on both sides of the LMMRE and connected with serpentine wires. The assembly was then packaged with elastic tape (3M4905) to maintain firm contact between the electrodes and the LMMRE while enhancing the elasticity of the sensing layer. Next, the sensing layer was secured inside the sensor housing with microscrews (M1.4) and covered. Finally, the bottom of the spherical cap was bonded to the sensing layer, with the convex surface oriented toward the patient's skin.

### Design of the LMMRE‐FES System

The system consists of a MyRio 1900 controller (National Instruments), an IMU module with a chip (WitMotion Shenzhen Co., Ltd.), an LMMRE sensor, and an electrical stimulation module. The sensor and IMU casings were high‐precision 3D‐printed components, designed in SOLIDWORKS and fabricated using a Bambu Lab 3D printer with transparent photosensitive resin. The system shell was constructed from acrylic sheets, also designed in SOLIDWORKS.

LMMRE sensors utilize the piezoresistive effect of LMMRE materials to generate signals. The signal acquisition circuit consists of a voltage divider resistor and an operational amplifier (AD9220), a standard configuration for extracting sensor signals. A voltage divider requires only a bias resistor to adjust the sensor's output voltage, with the resistor connected to ground (“pulled down”). An operational amplifier in a unity gain configuration buffers the output, which is then digitized using a signal acquisition device. In this study, a current‐voltage configuration is employed, where an operational amplifier and resistors control the output gain. The voltage across the sensor terminals remains constant, with the output voltage proportional to the current through the sensor. An analog‐to‐digital (AD) converter digitizes the analog signal and transmits it to the controller. The controller and host computer process the data using specialized algorithms to calculate real‐time muscle strength. This integrated architecture enables exceptional temporal response performance, achieving a total delay of only 25.35 ms from muscle force detection to electrical stimulation output. The system delay comprises three sequential stages: signal acquisition (10.05 ms), processing (10 ms), and output generation (5.3 ms), with hardware components accounting for 69% of the total latency and software processing contributing the remaining 31% (See Text S7, Supporting Information, for details).

### Patient Screening and Clinical Trial Protocol

This study evaluated the feasibility and therapeutic efficacy of FMG‐driven FES for upper limb rehabilitation through a controlled clinical trial. Eight stroke patients were recruited and randomly assigned to either an active training group or a passive control group. Rehabilitation outcomes were assessed at the trial's conclusion using standardized clinical metrics, including the Fugl‐Meyer Assessment, Brunnstrom Staging, ADL Scale, Ashworth Scale, and EMG analysis.

Participants (Table S1, Supporting Information) completed a 2‐week rehabilitation program, consisting of three 10‐min wrist‐raising exercises daily, with 5‐min rest intervals between sessions. In the active training group, motion intention was detected using a LMMRE sensor to control the FES system. Conversely, the control group underwent passive training with a pre‐set FES protocol lacking sensor integration. The pre‐set FES parameters were a frequency of 40 Hz and a total pulse width of 500µs, and the amplitude was adjusted according to the actual situation of patients (see details in Text S6, Supporting Information). Wrist‐lifting parameters were recorded using an IMU, ensuring precise monitoring of performance.

### Motor Function Reconstruction Experiment

Based on clinical trial scores, Subject 1 was selected to participate in the motor function reconstruction experiment. During the experiment, the subject sat on a soft chair or wheelchair with arms positioned horizontally on a cushioned table and forearms fully extended. Wrist elevation angles were measured using the IMU integrated into the LMMRE‐FES system. Before the experiment, the electrode sites were disinfected with a wet wipe or ethanol, and hydrogel electrode patches were applied to the affected side. During rehabilitation training, IMU data were collected simultaneously with LMMRE‐FES signals through hardware‐level synchronization (precision: ±0.2 ms). The main controller synchronized all data streams, enabling accurate post‐experimental correlation analysis.

During active training, LMMRE sensors were worn on the upper limbs, and the subject was instructed to lift the wrist to the maximum possible height. Wrist elevation was performed under three conditions for comparison: without FES, with FES operating under pre‐set parameters (no intent recognition), and with LMMRE‐FES. In the FES‐only condition, stimulation followed pre‐set parameters, whereas in the LMMRE‐FES condition, stimulation was guided by motor intent recognition (Movie S1).

### Statistical Analysis

Maximum angle distributions were statistically analyzed across three experimental conditions: no electrical stimulation, electrical stimulation, and LMMSE‐FES system application. IMU signals were captured via serial port and directly saved for analysis. Angular signals during movement were extracted from 800 cycles using the Findpeaks function in MATLAB, with results presented in Figure 5e using 2.5° statistical intervals. Individual variability in average maximum wrist elevation angles was assessed across four patients. Data are presented as mean ± standard error of the mean. One‐way analysis of variance (ANOVA) was performed to test for significant differences between patients, followed by Tukey's post hoc multiple comparison test. Different letter annotations (a, b, c, d) indicate statistically significant differences between groups (P < 0.05), as shown in Figure S15 (Supporting Information). All data curves, scatter plots, and distribution charts were generated using Origin2023 software.

### Ethics Approval Statement

Human and animal research in this study was approved by the Ethics Committee of the First Affiliated Hospital of the University of Science and Technology of China. The study is also registered with the Chinese Clinical Trial Registry (Registration No. ChiCTR2200066717).

## Conflict of Interest

The authors declare no conflict of interest.

## Author Contributions

Z.H. and Y.Y. contributed equally to this work. Z.H., Y.Y., and S.S. were responsible for the conceptualization of the study. Resources were provided by Z.H., Y.Y., X.Y., H.Z., L.X., S.Z., X.G., M.W., G.Y., and S.S. The methodology was developed by Z.H., Y.Y., G.Y., and S.S., while the investigation was carried out by Z.H., Y.Y., X.Y., H.Z., and L.X. Formal analysis was conducted by Z.H. and Y.Y. Supervision was provided by M.W., G.Y., and S.S. The original draft of the manuscript was written by Z.H. and Y.Y., with review and editing performed by M.W., G.Y., and S.S.

## Supporting information



Supporting Information

Supporting Information

## Data Availability

The data that support the findings of this study are available in the supplementary material of this article.
