# Supplementary material for: Upper Limb Motor Function Rehabilitation System Leveraging Pressure‐Sensitive Intent Recognition
Source: Adv Sci (Weinh). 2025 Jul 11;12(34):e05259. doi: 10.1002/advs.202505259 (PMC12442654; doi:10.1002/advs.202505259)
Supplement: Supplementary file 1 — Supporting Information [file ADVS-12-e05259-s002.docx]

Supplementary Information

- Upper Limb Motor Function Rehabilitation System Leveraging Pressure-Sensitive Intent Recognition

Zhiwei Hu, Yongchao Yin, Xiaoli Yang, Hanyang Zhang, Ling Xing, Shiwu Zhang, Xinglong Gong, Ming Wu^*^, Guolin Yun^*^, Shuaishuai Sun^*^

Z. Hu, L. Xing, S. Zhang, S. Sun

The First Affiliated Hospital of USTC

CAS Key Laboratory of Mechanical Behavior and Design of Materials

Institute of Humanoid Robots

Department of Precision Machinery and Precision Instrumentation

University of Science and Technology of China

Hefei, Anhui 230026, China

Email: [sssun@ustc.edu.cn](mailto:sssun@ustc.edu.cn)

Y. Yin, X. Gong, G. Yun

CAS Key Laboratory of Mechanical Behavior and Design of Materials

Department of Modern Mechanics

University of Science and Technology of China

Hefei, Anhui 230026, China.

Email: [ygl@ustc.edu.cn](mailto:ygl@ustc.edu.cn)

X. Yang, H. Zhang, M. Wu

The First Affiliated Hospital of USTC

Division of Life Sciences and Medicine

University of Science and Technology of China

Hefei, Anhui 230001, China.

Email: [Wumingkf@ustc.edu.cn](mailto:Wumingkf@ustc.edu.cn)

**This PDF file includes:**

Texts S1 to S10

Figures S1 to S18

Table S1 and S2

References

Supplementary Text

**Text S1. Conductive mechanism of composite materials.**

The sensing material of the sensor consists of piezoresistive composites, with their conductivity mechanism primarily explained by the tunneling effect theory ^[S1]^. (Supplementary references are designated with an 'S' prefix and listed separately in the Supplementary Materials section.) Rooted in quantum mechanics, this theory describes conductivity in piezoresistive materials through electron migration between conductive particles. In these composites, small changes in external loading induce significant variations in conductivity, thereby enhancing sensitivity. The conductive particles are closely spaced yet remain fully encapsulated within a polymer layer due to their distinct morphology.

The insulating polymer serves as a permanent separator between the particles, acting as the tunneling barrier. The quantum tunneling mechanism is facilitated by the unique structure of the conductive fillers, which possess sharp nanostructured tips or particles with very high aspect ratios. In the absence of mechanical deformation, the composite exhibits extremely high resistivity, comparable to the matrix material, and functions as an insulator. However, when the material is subjected to compression, stretching, or twisting, mechanical deformation reduces the thickness of the polymer layer between the conductive fillers. This reduction lowers the tunneling barrier, enabling the fillers to form a network of tunneling channels. The increased likelihood of tunneling phenomena significantly decreases the total resistance of the composite.

The fundamental unit in piezoresistive mathematical models is a tunneling structure formed by two particles separated by an insulating polymer layer. By modeling the conduction pathway as a chain of tunnel junctions, this approach extends the calculations to encompass the entire composite material. The current density between adjacent particles is an exponential function of their spacing, as described by the Fowler-Nordheim equation ^[S2]^:

$$\begin{aligned} J=\alpha\frac{{E_{V}}^{2}}{\phi}\exp\left( -\frac{\beta\phi^{\frac{3}{2}}}{E_{V}} \right)\#\left( S1 \right) \end{aligned}$$

where 𝛼 and 𝛽 are constants associated with the tunneling current, 𝜙 is the work function of the metal, $E_{V}$ is the electric field between neighboring particles, and 𝐽 is the current density formed between two particles.

This equation demonstrates that, for a given electric field strength, the current density is directly influenced by the spacing between neighboring particles. Consequently, the sensing material is highly responsive to variations in its thickness. Even slight changes in thickness caused by external pressure can result in ultra-high sensitivity.

**Text S2. Analysis of forces applied to inductive materials.**

For Region ①, where material deformation is predominantly governed by tensile action, the tensile strain can be characterized by examining the force distribution (Figure 2C):

$$\begin{aligned} \varepsilon_{1}=\frac{l^{'}-l}{l}=\frac{F_{n}}{E_{2}A_{2}}=\frac{F}{2\sin\theta E_{2}A_{2}}\#\left( S2 \right) \end{aligned}$$

where $l$ and $l'$ represent the original and deformed lengths of the material segment, respectively. $\theta$ denotes the angle between the deformed segment and substrate, and $F_{n}$ represents the tensile force acting on the deformed material, which can be decomposed into transverse ($F_{x}$) and longitudinal ($F_{y}$) force components. According to geometric relationship, the following expression can be established:

$$\begin{aligned} \cos\theta=\frac{l}{l^{'}}\#\left( S3 \right) \end{aligned}$$

Consequently, the relationship between $\varepsilon_{1}$ and $\theta$ can be derived:

$$\begin{aligned} \sin\theta=\frac{\sqrt{{\varepsilon_{1}}^{2}+2\varepsilon_{1}}}{\varepsilon_{1}+1}\#\left（ S4 \right） \end{aligned}$$

Substituting into Equation S2:

$$\begin{aligned} \varepsilon_{1}\frac{\sqrt{{\varepsilon_{1}}^{2}+2\varepsilon_{1}}}{\varepsilon_{1}+1}=\frac{F}{2E_{2}A_{2}}\#\left( S5 \right) \end{aligned}$$

This equation does not readily yield an exact analytical solution; therefore, we pursue an approximate solution. Given that material strain typically does not exceed 10% ^[48]^, the following condition is satisfied:

$$\begin{aligned} 0\leq\varepsilon_{1}\leq0.1\#\left( S6 \right) \end{aligned}$$

Under this constraint, the inequality is maintained:

$$\begin{aligned} \frac{1}{\varepsilon_{1}+1}\leq\frac{\sqrt{1+\frac{\varepsilon_{1}}{2}}}{\varepsilon_{1}+1}\leq\sqrt{1+\frac{\varepsilon_{1}}{2}}\#\left( S7 \right) \end{aligned}$$

Thus:

$$\begin{aligned} \frac{\sqrt{1+\frac{\varepsilon_{1}}{2}}}{\varepsilon_{1}+1}\subset\left( 0.91，1.025 \right)\#\left( S8 \right) \end{aligned}$$

Therefore, the relationship can be simplified to:

$$\begin{aligned} \varepsilon_{1}\frac{\sqrt{{\varepsilon_{1}}^{2}+2\varepsilon_{1}}}{\varepsilon_{1}+1}=\frac{\sqrt{2}{\varepsilon_{1}}^{\frac{3}{2}}\sqrt{1+\frac{\varepsilon_{1}}{2}}}{\varepsilon_{1}+1}\approx\sqrt{2}{\varepsilon_{1}}^{\frac{3}{2}}\#\left( S9 \right) \end{aligned}$$

Yielding the approximate solution for $\varepsilon_{1}$:

$$\begin{aligned} \varepsilon_{1}=\left( \frac{F}{2\sqrt{2}E_{1}A_{1}} \right)^{\frac{2}{3}}=\frac{1}{2}\left( \frac{F}{E_{1}A_{1}} \right)^{\frac{2}{3}}\#\left( S10 \right) \end{aligned}$$

For Region ②, where material deformation results from the combined effect of tensile and compressive forces, the longitudinal strain $\varepsilon_{2}$ comprises both tensile ($\varepsilon'$) and compressive ($\varepsilon''$) components. From Figure 2C, the force relationship can be expressed as:

$$\begin{aligned} F_{x}=\frac{F}{2\tan\theta}\#\left( S11 \right) \end{aligned}$$

$$\begin{aligned} F_{y}=\frac{F}{2}\#\left( S12 \right) \end{aligned}$$

Consequently, for the tensile and compressive strain components, the relationships are respectively:

$$\begin{aligned} \varepsilon^{'}=\frac{F_{x}}{E_{2}A_{2}}=\frac{F}{2\tan\theta E_{2}A_{2}}\#\left( S13 \right) \end{aligned}$$

$$\begin{aligned} \varepsilon^{''}=\frac{2F_{y}}{E_{1}A_{1}}=\frac{F}{E_{1}A_{1}}\#\left( S14 \right) \end{aligned}$$

According to Equation S3, the relationship between $\varepsilon_{1}$ and $\theta$ can be established:

$$\begin{aligned} \tan\theta=\sqrt{{\varepsilon_{1}}^{2}+2\varepsilon_{1}}\#\left( S15 \right) \end{aligned}$$

Incorporating Equation S15 and 4 yield a simplified expression for $\varepsilon^{'}$:

$$\begin{aligned} \varepsilon^{'}=\frac{1}{\sqrt{\left( \frac{E_{2}A_{2}}{F} \right)^{\frac{2}{3}}+4\left( \frac{E_{2}A_{2}}{F} \right)^{\frac{4}{3}}}}\#\left( S16 \right) \end{aligned}$$

Therefore, the final expression for the strain $\varepsilon_{2}$ in this region is:

$$\begin{aligned} \varepsilon_{2}=\nu\varepsilon^{'}+\varepsilon^{''}=\nu\frac{1}{\sqrt{\left( \frac{E_{2}A_{2}}{F} \right)^{\frac{2}{3}}+4\left( \frac{E_{2}A_{2}}{F} \right)^{\frac{4}{3}}}}+\frac{F}{E_{1}A_{1}}\#\left( S17 \right) \end{aligned}$$

where $\nu$ represents the Poisson's ratio of the material.

**Text S3. Simulation condition setting.**

We numerically simulated piezoresistive composites using a two-dimensional model to analyze the electric field distribution. Constructed in COMSOL, the model dimensions were 60×100μm, reflecting the microstructure of the material. It is important to note that while the two-dimensional simulation does not allow for a direct quantitative comparison with the volume resistivity of the actual three-dimensional composite structure, it does enable a qualitative assessment of resistivity changes under mechanical deformation.

For the composite, we chose a common conductive hybrid material system, which includes a polydimethylsiloxane (PDMS) matrix, liquid metal droplets (eGaIn), and iron (Fe) particles. The liquid metal droplets were modeled as incompressible fluids that deform in concert with the PDMS matrix, whereas the iron particles were treated as rigid circles with centroid positions that shift in response to the matrix deformation.

The process began with calculating the material's shape under stress, both pre- and post-deformation, based on the boundary conditions in the stress field. Subsequently, this geometric model was employed for electric field simulation. In these simulations, the two-dimensional model's right and left sides were electrically insulated, with the upper side grounded and the lower side set to a constant current density. The potential distribution of the sensing materials under various conditions was determined from the potential drop, as depicted in Figure S3. Furthermore, Figure 2h demonstrates the simulated current density distribution across the material's square cross-section. The comprehensive analysis of current density variations under different strain conditions (0%, 2%, 4%, 5%, 6%, and 8%) is presented in Figure S5. The minimal current density variation observed in the 0-4% strain range primarily reflects inherent limitations of the two-dimensional finite element model. Insufficient mesh refinement fails to capture microstructural changes under small strains, while limited mesh deformation cannot effectively simulate subtle adjustments in conductive particle spacing. Additionally, two-dimensional simulation cannot fully replicate the complex three-dimensional particle network dynamics within the composite material, hindering accurate representation of strain-induced contact resistance reduction and localized current density enhancement. However, beyond 4% strain, simulation results demonstrate significant nonlinear current density growth (161.2% increase, R²=0.98022), qualitatively validating the physical mechanism of strain-dependent resistance reduction. While finite element simulation cannot provide quantitative predictions, it effectively demonstrates the trend of reduced particle contact resistance and enhanced conductivity in LMMRE materials under substantial strain conditions.

**Text S4. Intention recognition algorithm formula derivation.**

LS-SVM Regression: The fundamental concept of the Support Vector Machine is to map the input data x from an inseparable low-dimensional feature space to a high-dimensional linearly separable feature space with the equation:

$$\begin{aligned} y=w^{T}\varphi\left( x \right)+b\#\left( S18 \right) \end{aligned}$$

where $\varphi$ is a nonlinear mapping function, $\omega$ and $b$ are the weight vector and bias term, respectively.

Thus, LS-SVM regression can be formulated as an equality constraint optimization problem:

$$\begin{aligned} \min_{w,b,e}J_{P}\left( w,e \right)=\frac{1}{2}w^{T}w+\frac{\gamma}{2}\sum_{k=1}^{N} e_{k}^{2}\#\left( S19 \right) \end{aligned}$$

subject to:

$$\begin{aligned} y_{k}=w^{T}\varphi\left( x_{k} \right)+b+e_{k},k=1,\ldots,N\#\left( S20 \right) \end{aligned}$$

where $\gamma$ is the weight determining the trade-off between minimizing training error and finding the optimal hyperplane, $e_{k}$​ is the slack variable, and $N$ is the total number of training data. The optimization problem is solved using the Lagrange multiplier method:

$$\begin{aligned} L\left( w,b,e;\alpha\right)=J_{P}\left( w,e \right)-\sum_{k=1}^{N} \alpha_{k}\left( w^{T}\varphi\left( x_{k} \right)+b+e_{k}-y_{k} \right)\#\left( S21 \right) \end{aligned}$$

where $\alpha_{k}$ is the Lagrange multiplier. By setting the partial derivatives of $\omega$,$b$,$e_{k}$and $\alpha_{k}$ ​ to zero, a system of linear equations is obtained:

$$\begin{aligned} \left. \left[ \begin{matrix} 0 & 1_{v}^{T} \\ 1_{v} & \Omega+\frac{I}{\gamma} \end{matrix} \right. \right]\left[ \begin{aligned} b \\ \alpha\end{aligned} \right]=\left[ \begin{aligned} 0 \\ y \end{aligned} \right]\#\left( S22 \right) \end{aligned}$$

$$\begin{aligned} \Omega=\varphi(x_{k})^{T}\varphi(x_{l})=K(x_{k},x_{l}),k,l=1,\ldots,N\#\left（ S23 \right） \end{aligned}$$

where $1_{v}$ is the N-dimensional vector of ones, $I$ is the N-dimensional identity matrix, and $\alpha=\left[ \alpha_{1},\ldots,\alpha_{N} \right]^{T}.K\left( x_{k},x_{l} \right)$ is the kernel function, and the LS-SVM regression model is defined as:

$$\begin{aligned} y\left( x \right)=\sum_{k=1}^{N} \alpha_{k}K\left( x,x_{k} \right)+b\#\left（ S24 \right） \end{aligned}$$

The control algorithm is implemented using LabVIEW software and runs on the host computer and MyRio controller. The algorithm consists of three main functional modules: data acquisition, algorithm processing, and output control. The data acquisition module operates in the FPGA environment and collects data from the AD converter through the digital I/O interface. This module provides a sampling frequency of 40MHz using the onboard clock of the MyRio. The digital I/O interface includes 12 channels that can output 12-bit voltage amplitudes between 0 and 5V. The algorithm module is responsible for processing the data collected by the data acquisition module. It consists of three parts: signal filtering, smoothing, and intent recognition. The signal is filtered through a Butterworth low-pass filter to suppress noise (cutoff frequency of 5 Hz), removing high-frequency signals. After filtering, the signal is smoothed using a moving average with a 100-millisecond window to achieve a smooth signal level.

**Text S5. Assessment tools for post-stroke rehabilitation: An overview of the FMA, Brunnstrom Staging, ADL, and Ashworth Scale.**

The Fugl-Meyer Assessment is a widely used clinical tool for evaluating motor function in post-stroke patients. It assesses the upper and lower extremities, balance, sensation, and joint range of motion. Each item is scored on a scale from 0 to 2, where 0 denotes no movement or function, 1 indicates partial movement or function, and 2 represents complete movement or function. Reflexes are scored on a binary scale (0 or 2). The maximum possible FMA score for the upper extremities is 66, reflecting full recovery of both sensory and motor functions.

The Brunnstrom Staging is a staging system utilized to describe the various stages of motor function recovery in patients with hemiplegia following a stroke. This system aids therapists in assessing the patient's progress and in formulating an appropriate treatment plan. The six stages of Brunnstrom assessment include: the delay stage, spasticity stage, synergistic voluntary movement stage, partial synergistic voluntary movement stage, synergistic voluntary movement stage, and the coordinated voluntary movement stage. The coordinated voluntary movement stage indicates that motor function is nearly normal.

The Activities of Daily Living (ADL) score is a suite of tools employed to assess an individual's capacity for self-care in their daily life. It typically encompasses two main components: basic activities of daily living and instrumental activities of daily living. This assessment method is straightforward, reliable, and sensitive, making it suitable for detecting changes in independent living activities before and after treatment. The total score for the basic activities of daily living scale used in this study is 100 points, with higher scores indicating greater independence and less dependence.

The Ashworth Scale is a tool designed to assess the degree of muscle spasticity and is frequently used in patients with neurological conditions such as stroke, spinal cord injury, multiple sclerosis, cerebral palsy, and brain trauma. This scale grades spasticity by evaluating muscle resistance during passive movement. In this study, the Modified Ashworth Scale (MAS) was utilized, which introduces a 1+ level to the original scale to enhance the sensitivity of the assessment. The improved Ashworth Scale transforms the assessment of spasticity from a qualitative to a quantitative measure, thereby increasing the precision of the assessment. A lower rating on this scale indicates that the patient is closer to the normal level.

**Text S6. Patient screening and clinical trial protocol details.**

This study conducted a controlled clinical trial to evaluate the feasibility and therapeutic efficacy of FMG-driven FES for upper limb active training. The trial received approval from the Ethics Committee of the First Affiliated Hospital of the University of Science and Technology of China. Eight stroke patients meeting the inclusion criteria were recruited, and written informed consent was obtained prior to eligibility assessment. Participant demographic data are summarized in Table S1. Patients were randomly assigned to either the active training group or the passive training control group before rehabilitation commenced. All participants underwent evaluations at the conclusion of the trial. Training outcomes were assessed using various clinical methods, including the Fugl-Meyer Assessment, Brunnstrom Staging, ADL, Ashworth Scale, and EMG evaluation.

Table S1．Demographic characteristics of patients.

| **Patient** | **Group** | **Gender** | **Age (years)** | **Stroke types** | **Lesion side** | **Time since stroke (month)** |
| --- | --- | --- | --- | --- | --- | --- |
| 1 | A | F | 43 | Ischemic | Left | 19 |
| 2 | A | F | 51 | Hemorrhagic | Right | 1 |
| 3 | A | M | 60 | Ischemic | Left | 1 |
| 4 | A | F | 67 | Ischemic | Left | 1 |
| 5 | P | M | 38 | Ischemic | Left | 1 |
| 6 | P | M | 49 | Ischemic | Right | 12 |
| 7 | P | M | 65 | Ischemic | Left | 1 |
| 8 | P | M | 55 | Ischemic | Left | 1 |

A stands for active group, P stands for passive group, M stands for male, F stands for female.

The developed upper limb movement rehabilitation system (LMMRE-FES) required no initial training, and all participants underwent clinical safety evaluations. During the two-week training period, subjects received daily rehabilitation therapy. In the active training group, electrodes were placed on the affected side, and sensors were installed on the upper limb to detect motion intention and control the FES system. Participants were divided into ipsilateral and contralateral active training groups based on muscle strength. For Brunnstrom stages I and II, sensors were placed on the healthy side to enable contralateral intent recognition, while for Brunnstrom stage III and above, sensors were placed on the affected side for ipsilateral active training. The control group underwent passive training with a pre-set FES program and no sensor integration.

Subjects performed three sets of 10-minute rehabilitation exercises daily, with five-minute rest periods between sets. Skin at the electrode site was disinfected with ethanol or a wet paper towel before applying hydrogel electrode patches. Each experiment required a new hydrogel electrode. The FES system promoted active movement, with the active training group tasked to lift their wrists while seated. Wrist height was recorded using an inertial measurement unit (IMU), and the FES system adjusted output current based on individual responses. FES parameters^[S3]^ were set to a frequency of 40 Hz(selected because frequencies below 20 Hz induce irregular muscle contractions while frequencies above 50 Hz readily cause muscle fatigue), a total pulse width of 500 μs (chosen to effectively stimulate motor neurons within the 150-250 μs range, balancing therapeutic efficacy and patient comfort), current amplitudes tailored to wrist angle and muscle response (individually adjusted to the minimum effective intensity that produces significant wrist extension without causing pain), and a symmetrical biphasic square wave pulse (preferred over monophasic waveforms to reduce electrode polarization effects and minimize tissue damage risk). The LMMRE sensor detected motor intent and activated the FES system accordingly. For the control group, the FES system delivered timed electrical pulses with pre-set parameters and identical current amplitudes. Therapists monitored participants, intervening only in cases of equipment failure or discomfort. Participants were instructed to avoid unnecessary movements, talking, or distractions and were required to maintain motor attempts during FES stimulation until full hand extension was achieved. Trials were suspended if participants experienced pain, fatigue, or an inability to maintain a safe posture. After completing the two-week treatment, rehabilitation outcomes were assessed and compared to pre-treatment evaluations.

In this experiment, subjects were evaluated using electromyography (EMG) before and two weeks after treatment with a biofeedback therapy instrument (S4-30, VISHEE Nanjing). During the evaluation, subjects sat in a chair with their forearms placed horizontally on a table. After attaching hydrogel electrodes, the biofeedback device recorded EMG signals as the subjects performed five wrist-raising movements without electrical stimulation.

Additionally, subject 1 and subject 2 underwent functional near-infrared spectroscopy (fNIRS) testing to evaluate brain activation during LMMRE-FES treatment. After a 15-minute resting period to stabilize HbO levels, the subjects performed a 20-minute wrist-lifting task, identical to the task described earlier. Brain function activation was assessed using a multi-channel NirScan near-infrared brain imaging system (Danyang Huichuang Medical Equipment Co., Ltd.). Functional connectivity maps between brain regions were generated by processing effective segmented data through block averaging and model building.

**Text S7. Signal delay analysis of the LMMRE-FES rehabilitation system.**

The LMMRE-FES system exhibits a total delay of approximately 25.35 ms, comprising three primary components: signal acquisition (10.05 ms), processing (10 ms), and output generation (5.3 ms). Hardware elements contribute approximately 69% of the total latency, with ADC sampling representing the principal bottlenecks (10 ms). Software processing accounts for approximately 31% (10 ms) of the total delay, primarily arising from LS-SVM algorithm-based intention recognition and dynamic FES parameter adjustment. The hardware system excluding the sensor demonstrates a cumulative delay of approximately 5.3 ms, constituting 20.9% of the overall system latency. Under stable operating conditions, individual components exhibit delay characteristics of varying magnitudes. The LCC120 optocoupler's signal isolation transmission (5 ms) represents the primary bottleneck, while the LM358 operational amplifier operates at sub-microsecond levels (292 ns) and IRF series MOSFET switching times fall within the nanosecond range (42-120 ns). Power management devices RY3710 and MAX1771 contribute minimal latency under steady-state conditions (<5 ns), inducing only transient effects ranging from microseconds to milliseconds during sudden load variations. In clinical rehabilitation contexts, the system's 25.35 ms delay satisfies real-time requirements for muscle surface pressure intention detection to FES stimulation output, substantially below the natural reaction time^[S4]^ of human neuromuscular systems (approximately 100 ms), thereby ensuring system effectiveness during rehabilitation training.

**Text S8. Performance comparison experiments with commercial piezoresistive sensor**

Comprehensive performance evaluation comparing the LMMRE sensor with a commercial piezoresistive sensor (IMS-C10A) demonstrates superior reliability across multiple clinically relevant metrics (Figures 2e, S18-S19). Extended reliability testing over 1000 loading cycles (>1 hour duration) revealed exceptional long-term stability of the LMMRE sensor, with output voltage variations remaining below 5% throughout the test period (Figure 2e), compared to approximately 20% drift exhibited by commercial piezoresistive sensors within comparable timeframes according to the datasheet. Cross-sensitivity analysis further demonstrates exceptional interference rejection under tangential loading, with the LMMRE sensor maintaining signal stability (<5% variation) while the commercial sensor exhibited dramatic fluctuations of 98.4%, 429.4%, and 570.1% under 50g, 100g, and 150g lateral forces, respectively (Figure S18a). Temperature sensitivity evaluation over 250 seconds confirmed the LMMRE sensor's thermal stability (3.49% drift) compared to the commercial sensor (13.80% drift), indicating superior performance consistency across environmental variations (Figure S18b). Both sensors showed comparable minimal response to simulated physiological saline exposure, with variations of 0.92% (LMMRE) versus 0.88% (commercial sensor) (Figure S18c). Dynamic skin-contact stability assessment during 100 consecutive wrist elevation cycles demonstrated the LMMRE sensor's superior interface reliability, maintaining only 4.3% variation in average peak amplitude between initial and final measurement cycles, compared to 8.7% degradation observed with the commercial sensor (Figure S19). These comprehensive results confirm the LMMRE sensor's enhanced robustness against critical performance-limiting factors—long-term drift, tangential forces, temperature fluctuations, and skin-contact variations—that compromise reliability in rehabilitation monitoring applications. The superior multi-dimensional performance directly translates to improved signal fidelity and reduced false triggering in clinical functional electrical stimulation systems.

**Text S9. Explanation of Follow-up Data.**

To evaluate the sustainability of therapeutic improvements, participants underwent structured follow-up assessments at 3 months post-intervention using identical clinical evaluation protocols: Brunnstrom Staging, Activities of Daily Living (ADL) Scale, and Modified Ashworth Scale. Participants maintained their standard post-discharge rehabilitation routines and reported any significant clinical changes or additional interventions during follow-up. Data collection employed the same management protocols established for the initial trial. As demonstrated in Table S2, participants exhibited sustained functional improvements across all three assessment scales.

Table S2．The follow-up data of the patients three months later.

| **Patient** | **Group** | **Gender** | **Age (years)** | **Brunnstrom**  **Stage** | **ADL** | **Ashworth** |
| --- | --- | --- | --- | --- | --- | --- |
| 1 | A | F | 43 | VI | 97 | 0 |
| 2 | A | F | 51 | VI | 99 | 0 |
| 3 | A | M | 60 | V | 58 | 0 |
| 4 | A | F | 67 | III | 56 | 0 |
| 5 | P | M | 38 | III | 84 | 1+ |
| 6 | P | M | 49 | III | 80 | 1 |
| 7 | P | M | 65 | V | 73 | 1 |
| 8 | P | M | 55 | III | 67 | 0 |

A stands for active group, P stands for passive group, M stands for male, F stands for female.

**Text S10. Comparison with EMG-driven electrical stimulation system.**

Comparative analysis through literature review, theoretical evaluation, and experimental validation demonstrates distinct advantages of the LMMRE-FES system relative to conventional EMG-driven FES approaches. The LMMRE-FES system employs liquid metal-based mechanoresistive sensing that detects muscle activity through physical deformation, fundamentally differing from EMG systems that measure electrical potentials during muscle activation. Simultaneous testing revealed synchronized activation patterns between LMMRE-FES and EMG-driven systems during repetitive wrist elevation tasks, with both modalities demonstrating comparable sensitivity and responsiveness across five consecutive cycles over a 16-second recording period (Figure S16). While EMG signals can detect neuromuscular intention prior to visible movement, they are inherently susceptible to electromagnetic interference, crosstalk from adjacent muscle groups, and skin impedance variations^[S5,S6]^. Conversely, the LMMRE-FES provides mechanical specificity that mitigates erroneous activation in multi-muscle regions such as the forearm. EMG systems require sophisticated signal processing algorithms, precise electrode positioning, and frequent recalibration during prolonged use due to electrode impedance fluctuations. The LMMRE-based approach offers enhanced calibration stability and reduced recalibration requirements in clinical settings through its mechanical sensing modality. This comparative analysis confirms effective motor intent detection capabilities of force myography (FMG) with unique advantages for rehabilitation applications.


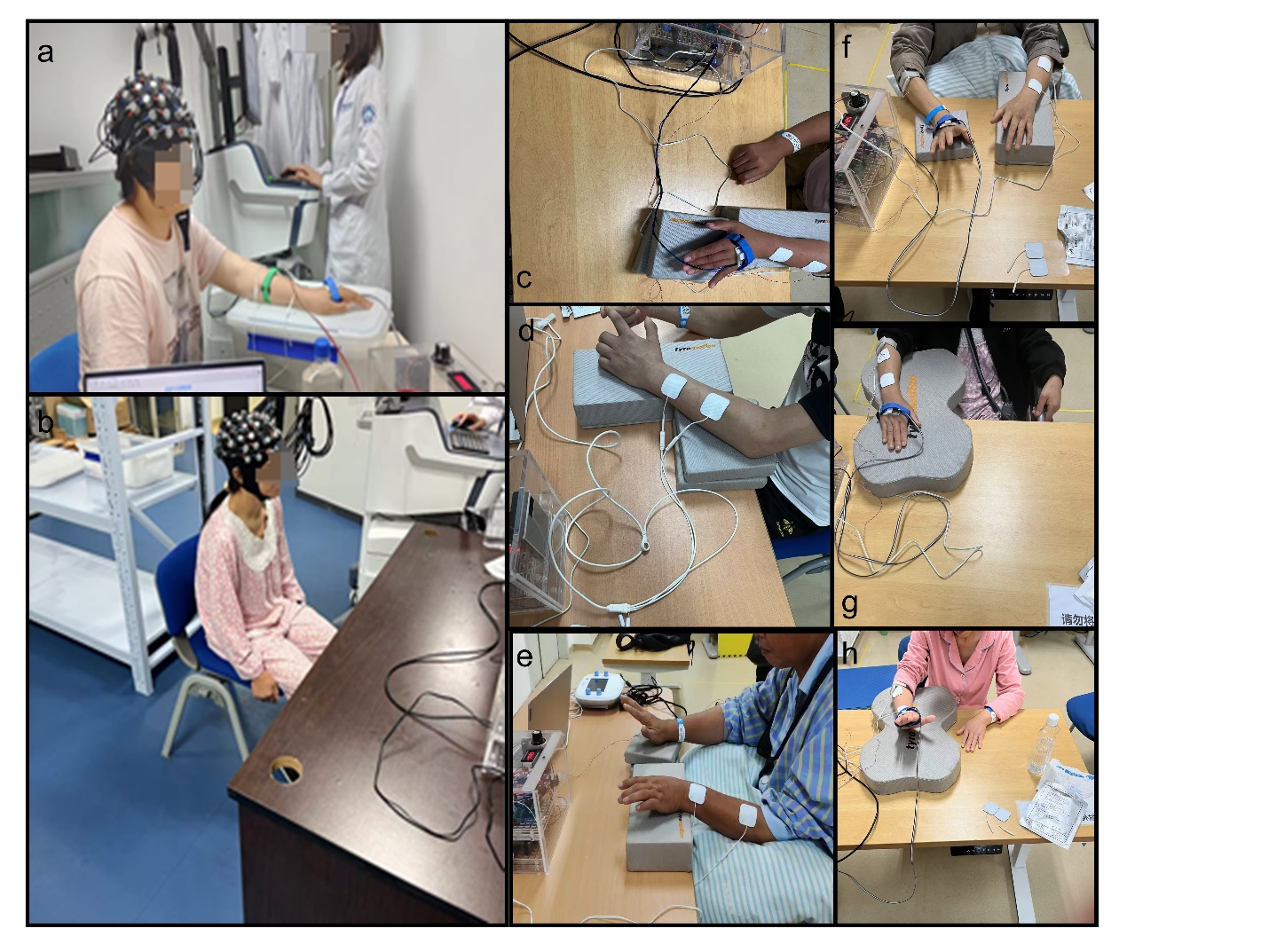


Figure S1. The group photograph of the patient test. (a, b) fNIRS tests. (c, d, e) Control group experiment. (f, g, h) Active training group experiment.

These photographs encompass FMG acquisition, electrical stimulation parameters selection, ankle data recording, LMMRE-FES system verification, brain oxygen tests, and various other assessments.


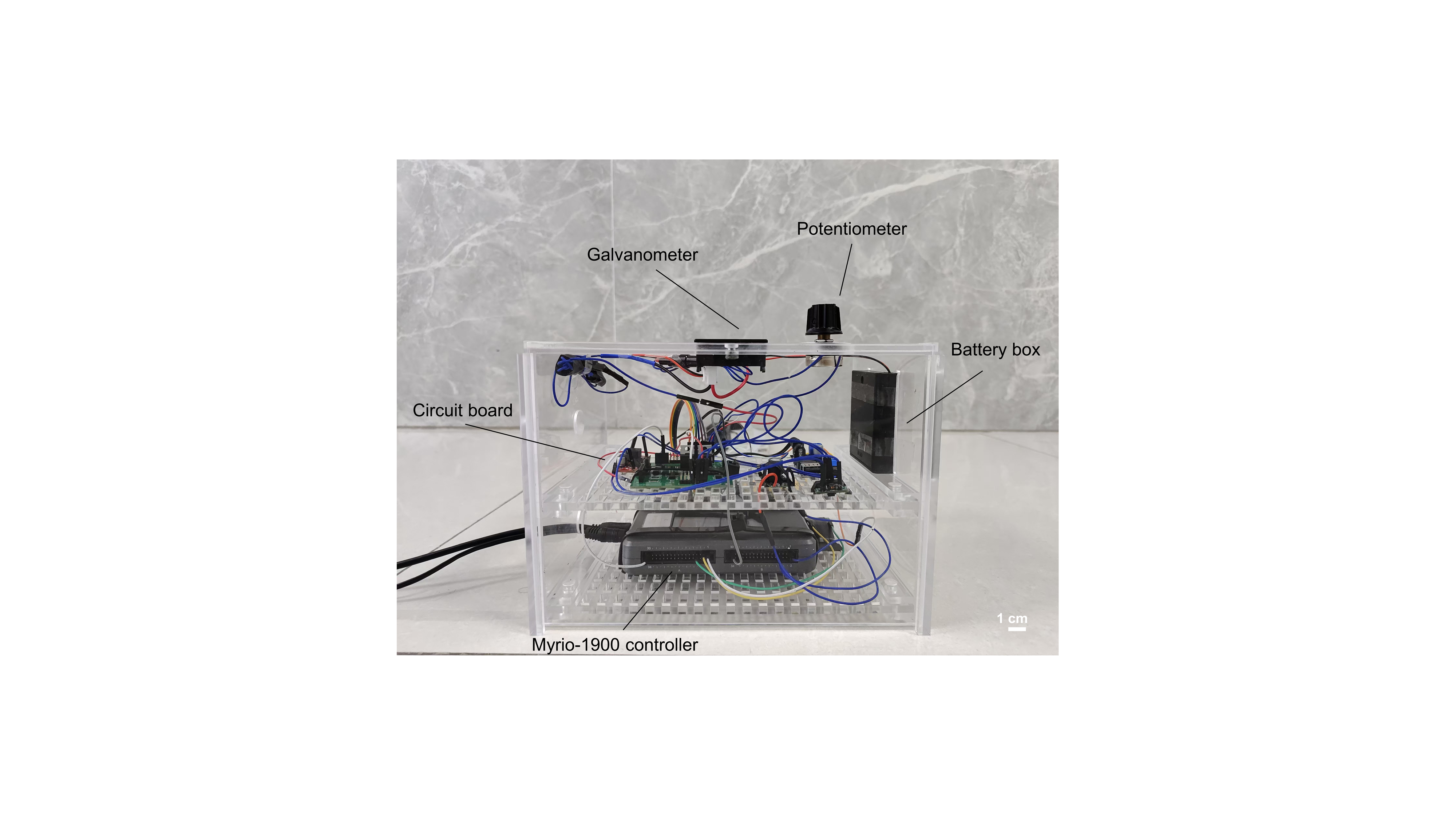


Figure S2. System Hardware Physical Diagram.

The system hardware includes a galvanometer, battery box, circuit board, potentiometer, and the Myrio-1900 controller, among other components. Each element is integrated to ensure precise functionality and efficient performance within the system.


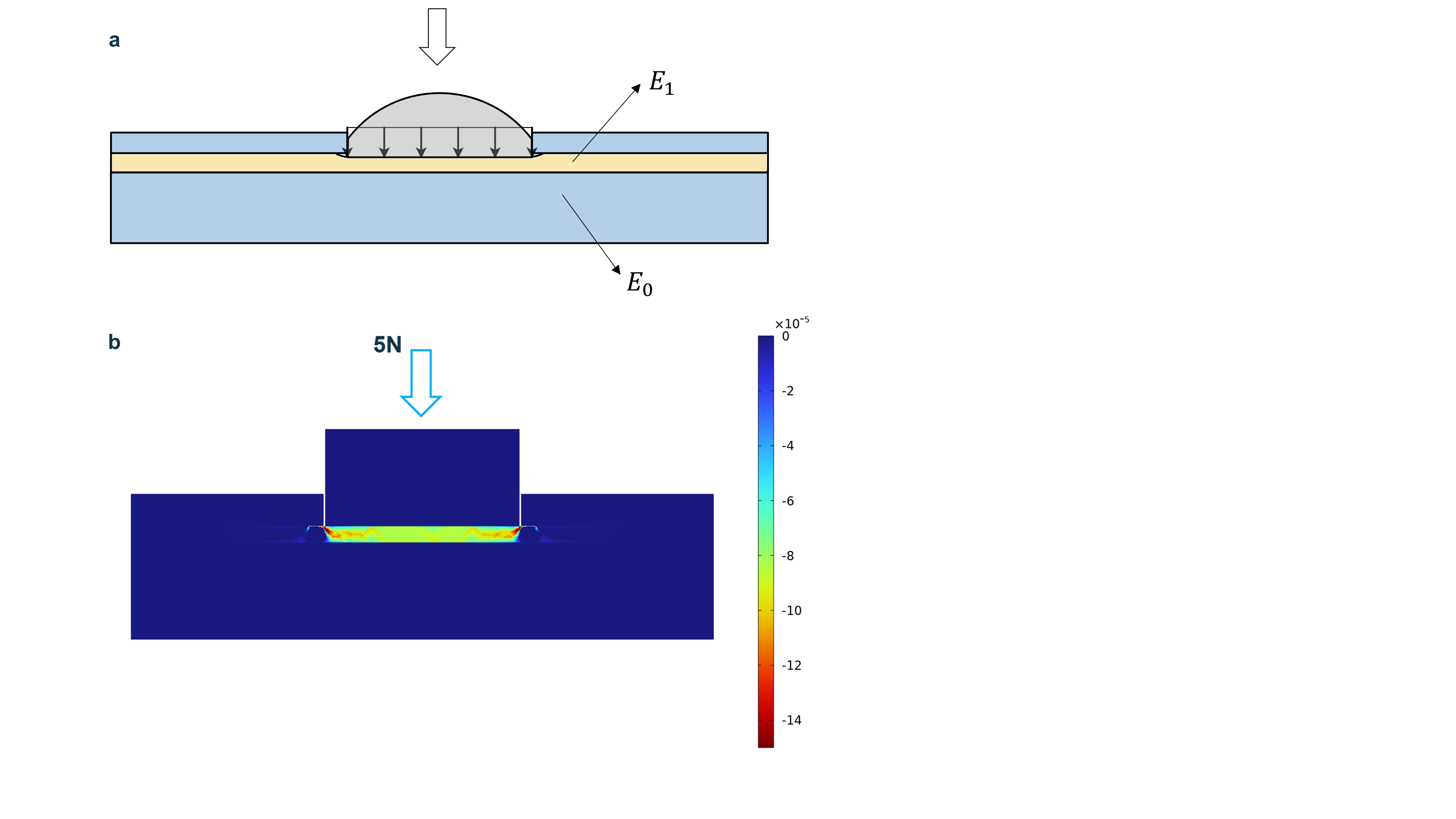


Figure S3. a) A schematic diagram of the traditional sandwich structure. b) Simulation diagram of the traditional sandwich structure.


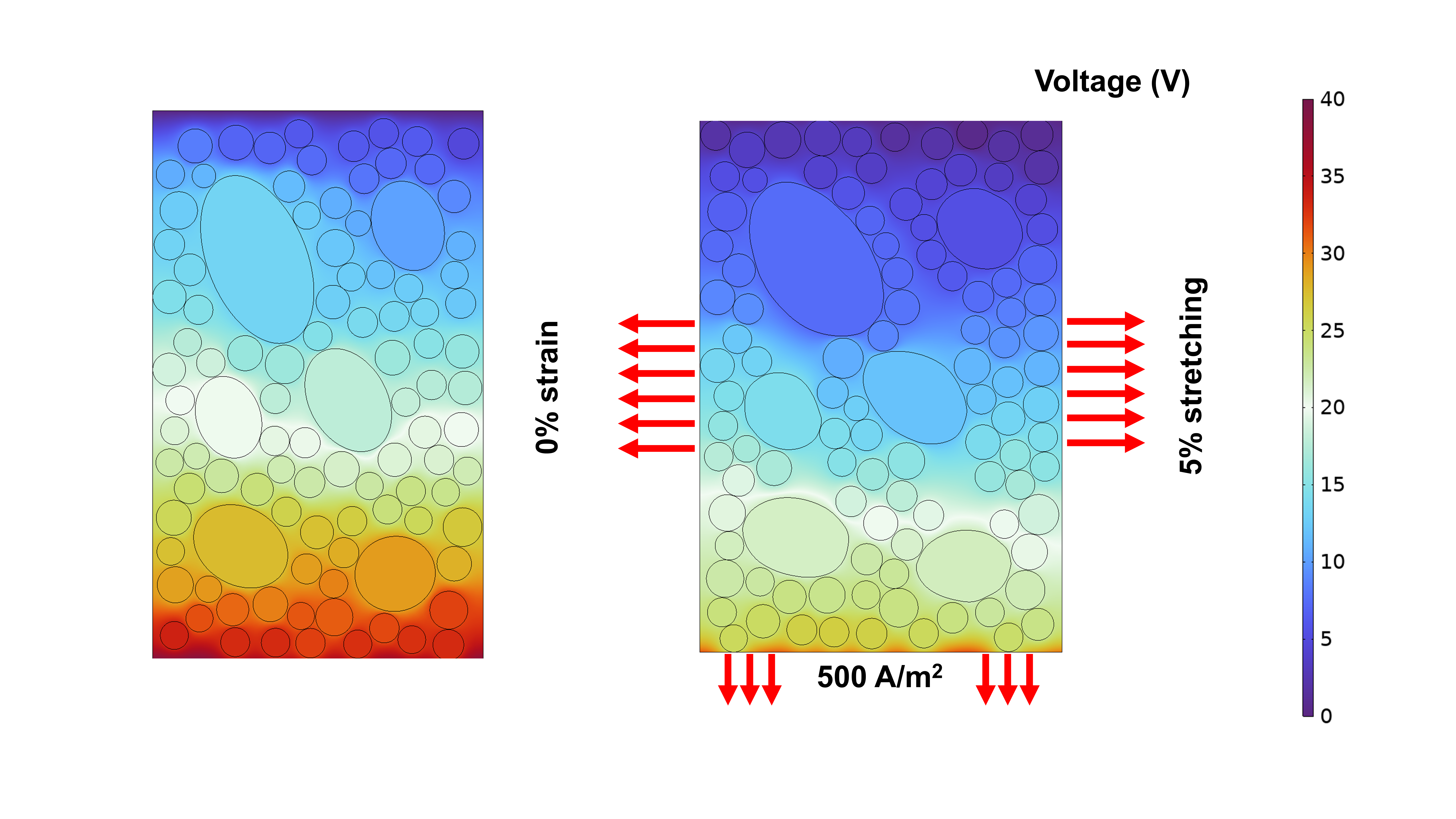


Figure S4. Simulation of potential distribution before and after deformation of sensing material.

In the simulation, the left and right sides of the two-dimensional model are electrically insulated, the upper side is grounded, and the current density at the lower side is fixed. The potential distribution of the sensing material under varying conditions is calculated based on the potential drop. The color gradient, ranging from blue to red, represents this distribution. A more pronounced gradient indicates higher resistance in the thin layers of the composite material. The simulation results clearly show that, as the material deforms, the color gradient decreases, signifying a reduction in resistance. This trend aligns with the current density simulation results, further supporting the qualitative analysis of how mechanical deformation influences the material’s resistivity.


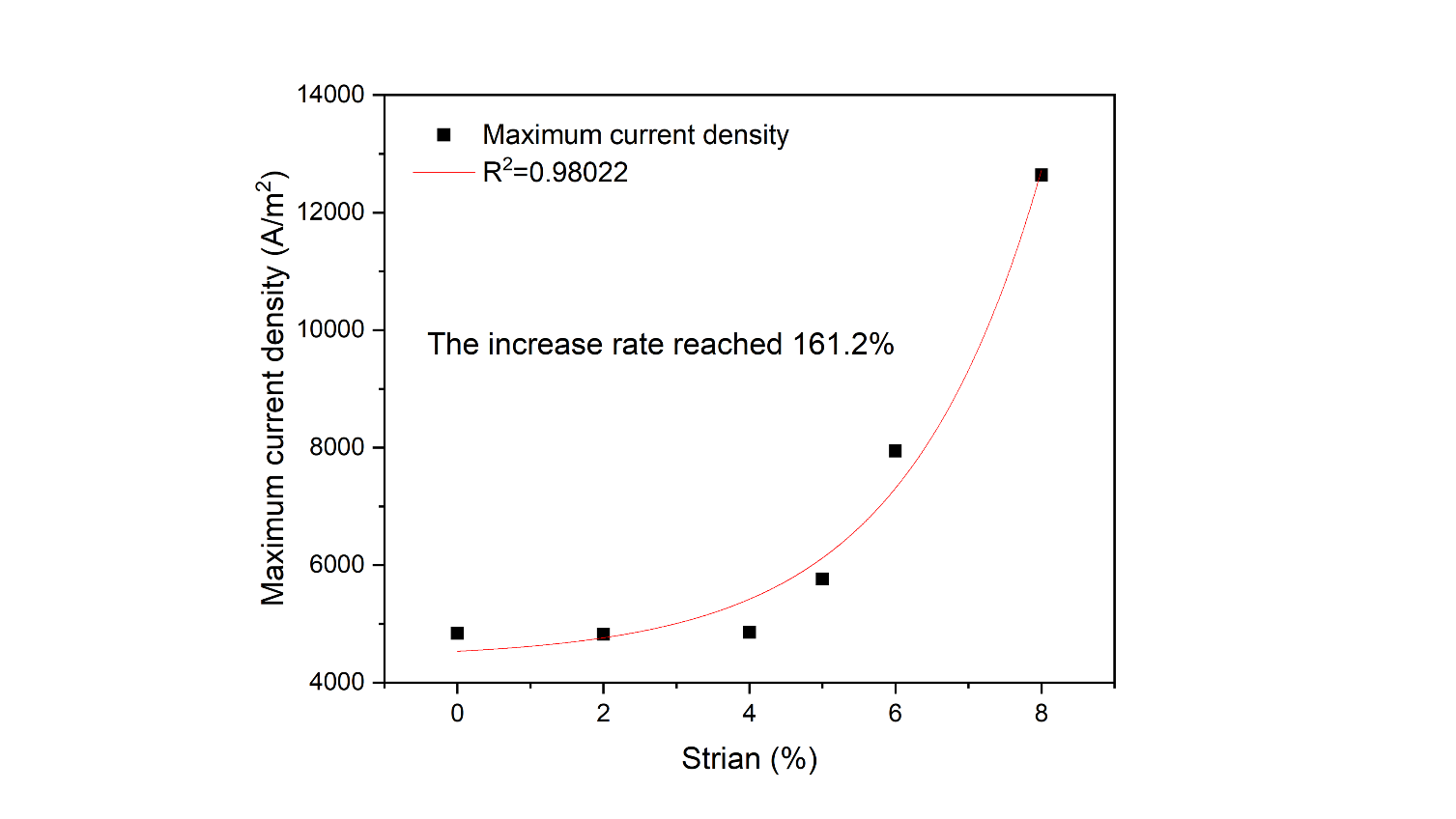


Figure S5. Maximum current density in different strain.

The figure demonstrates the strain-dependent maximum current density response of the LMMRE material. At low strain levels (0-4%), the sensor maintains a stable baseline response, transitioning to dramatic exponential growth under higher deformations. The exponential fit (R² = 0.98022) accurately captures the nonlinear response characteristics, with current density increasing from 4840 A/m² at baseline to 12640 A/m² at 8% strain—representing a substantial 161.2% enhancement.


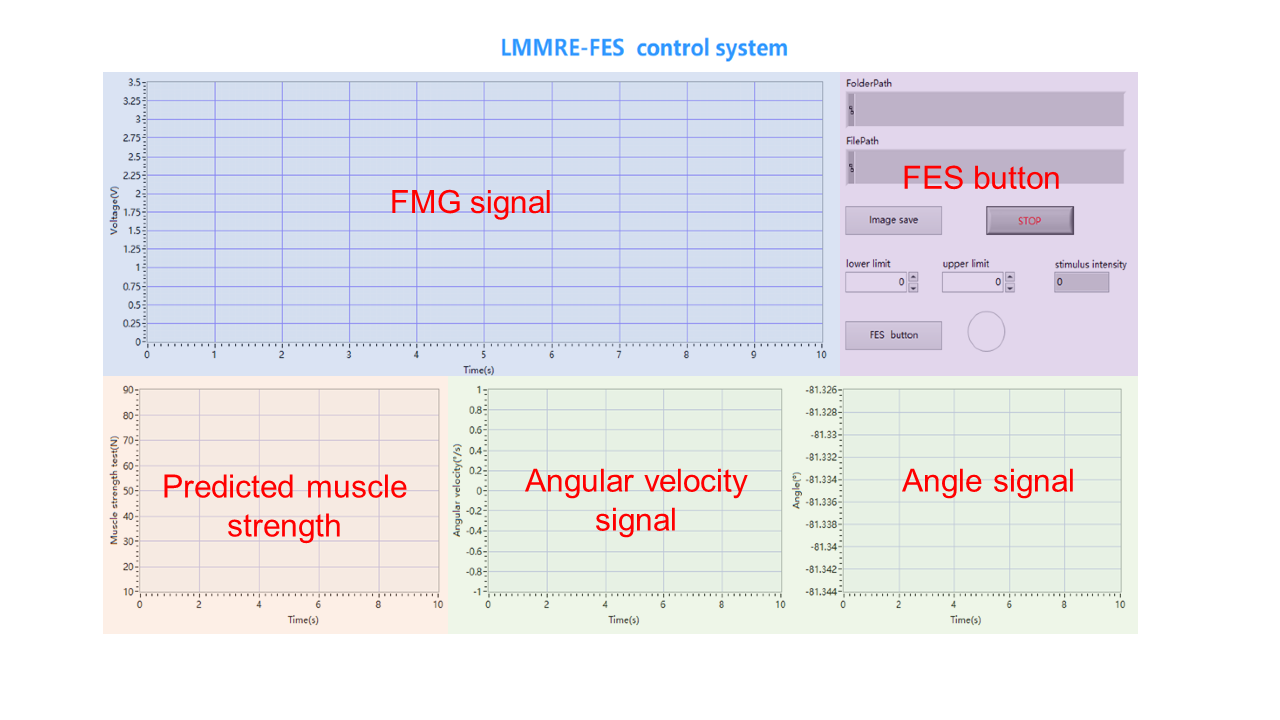


Figure S6. Upper Computer Control Interface.

The upper computer control interface is designed to display FMG signals, joint angles, angular velocity, and predicted muscle strength in real time. It enables precise regulation of the functional electrical stimulation output based on the FMG signal, ensuring dynamic and adaptive control for optimal system performance.


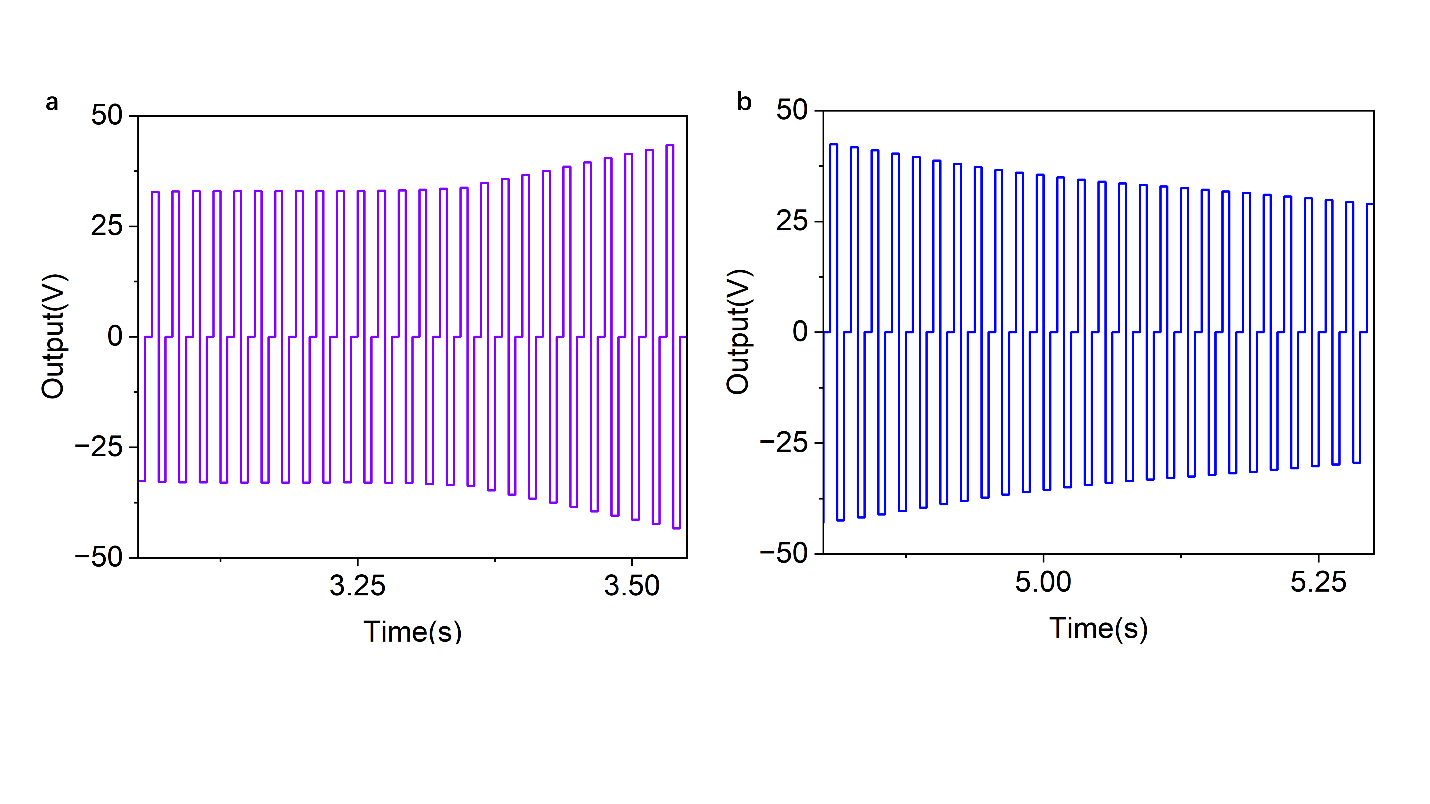


Figure S7. The modulation waveform corresponding to the (a) increasing and (b) decreasing sections of FMG.


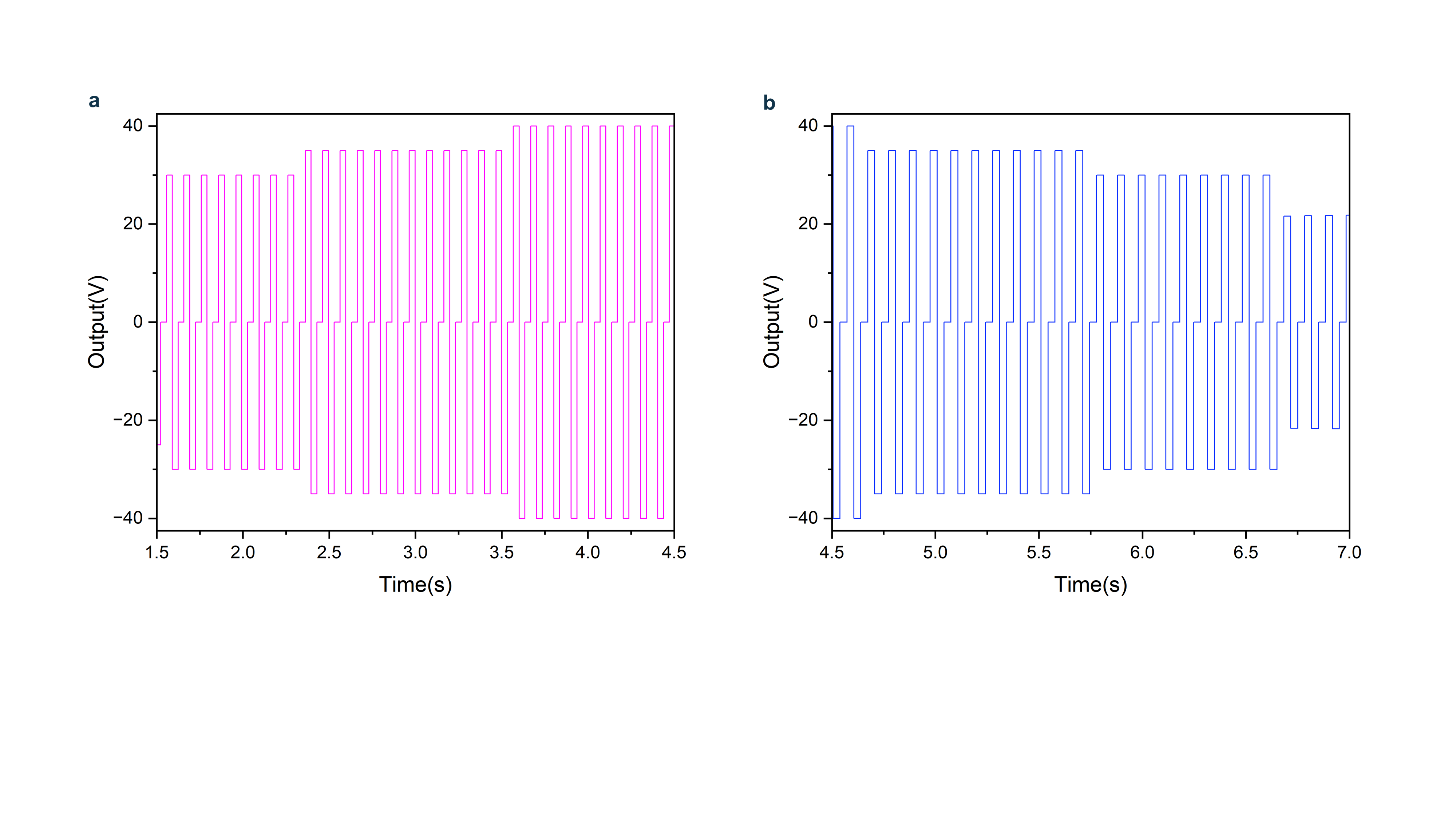


Figure S8. The modulation waveform corresponding to the (a) increasing and (b) decreasing sections of traditional FES (manual adjustment).


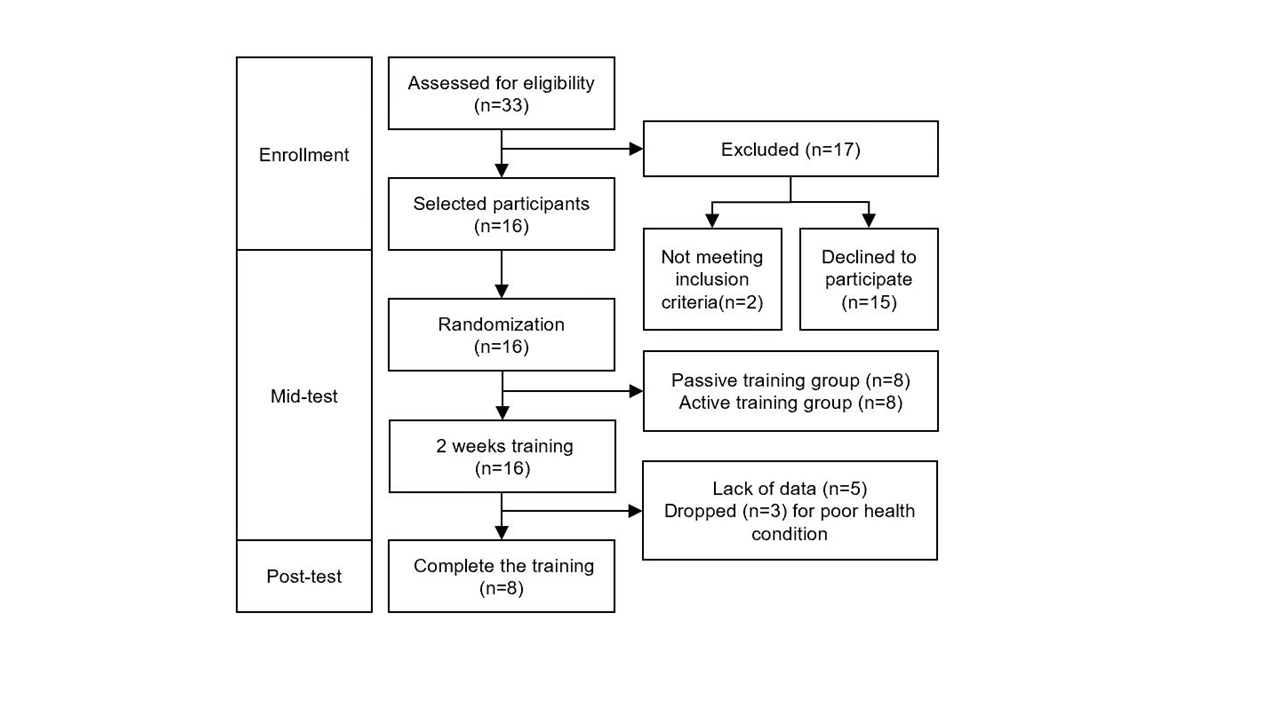


Figure S9. Subject screening procedures.

This diagram illustrates the participant flow in the study. Initially, 33 participants were assessed for eligibility, of whom 17 were excluded: 2 did not meet the inclusion criteria, and 15 declined to participate. As a result, 16 participants were enrolled and randomly assigned to two groups: 8 to the passive training group and 8 to the active training group. All participants underwent a two-week training program. During the post-training phase, 8 participants completed the entire training and were tested. However, 5 participants were unable to complete the study due to missing data, while 3 others dropped out for health reasons.


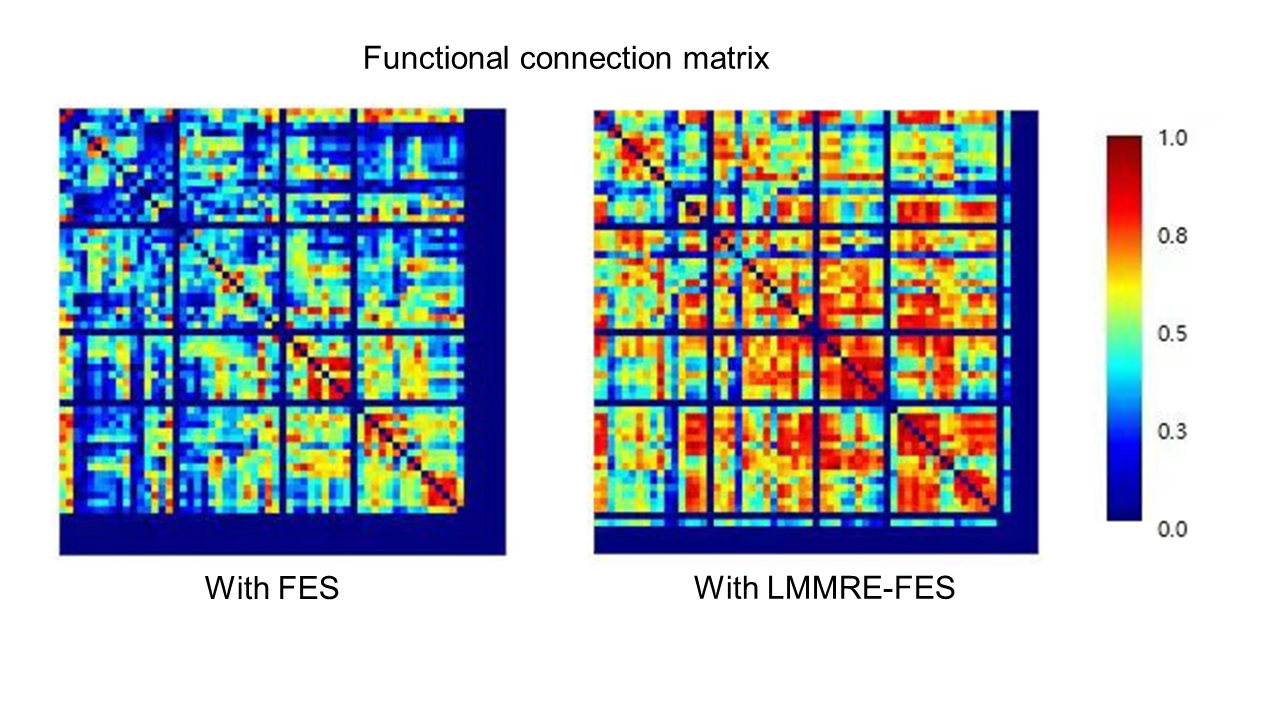


Figure S10. Functional connection matrix of fNIRS data.

This diagram illustrates the functional connectivity matrix under two conditions: "With FES" and "With LMMRE-FES". The left matrix predominantly shows blue and yellow, indicating low correlation between signals and weak functional connectivity. In contrast, the right matrix features broader red areas, signifying stronger correlations and significantly enhanced functional connectivity. This shift suggests that LMMRE-FES actively promotes functional connectivity between brain regions, facilitating closer interactions between different signal sources. These findings support the potential of LMMRE-FES to improve functional connectivity in brain regions.


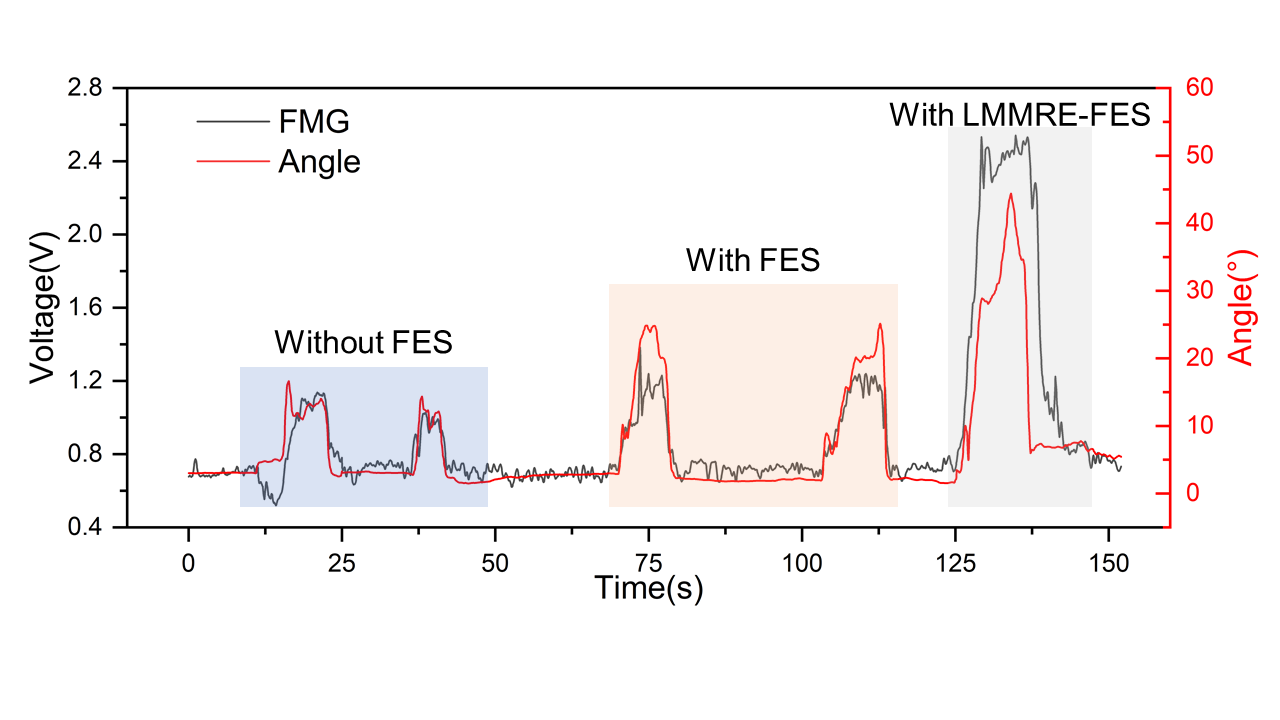


Figure S11. Changes of FMG and angle in motor function reconstruction experiments.

The three areas represent the three experimental conditions: unassisted, single FES, and LMMRE-FES.


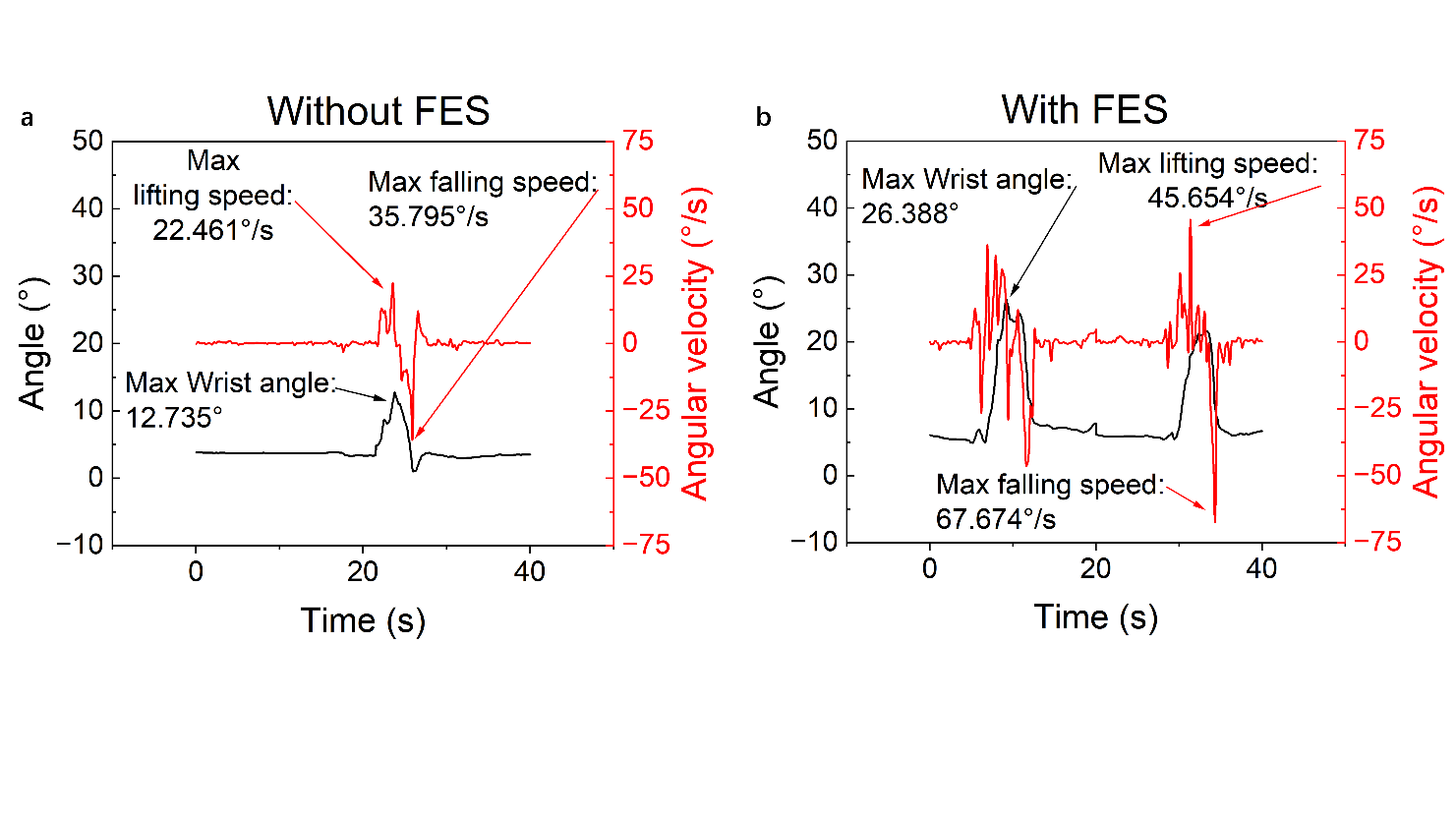


Figure S12. Changes of angle and angular velocity in motor function reconstruction experiments. a Changes in angle and angular velocity of wrist lift in patient without FES. b Changes in angle and angular velocity of wrist lift in patient with single FES.

The figure illustrates the changes in the patient's corresponding angle and angular velocity under two conditions: without assistance and with FES. It also shows the maximum wrist angle, maximum lifting angular velocity, and maximum falling angular velocity for each condition.


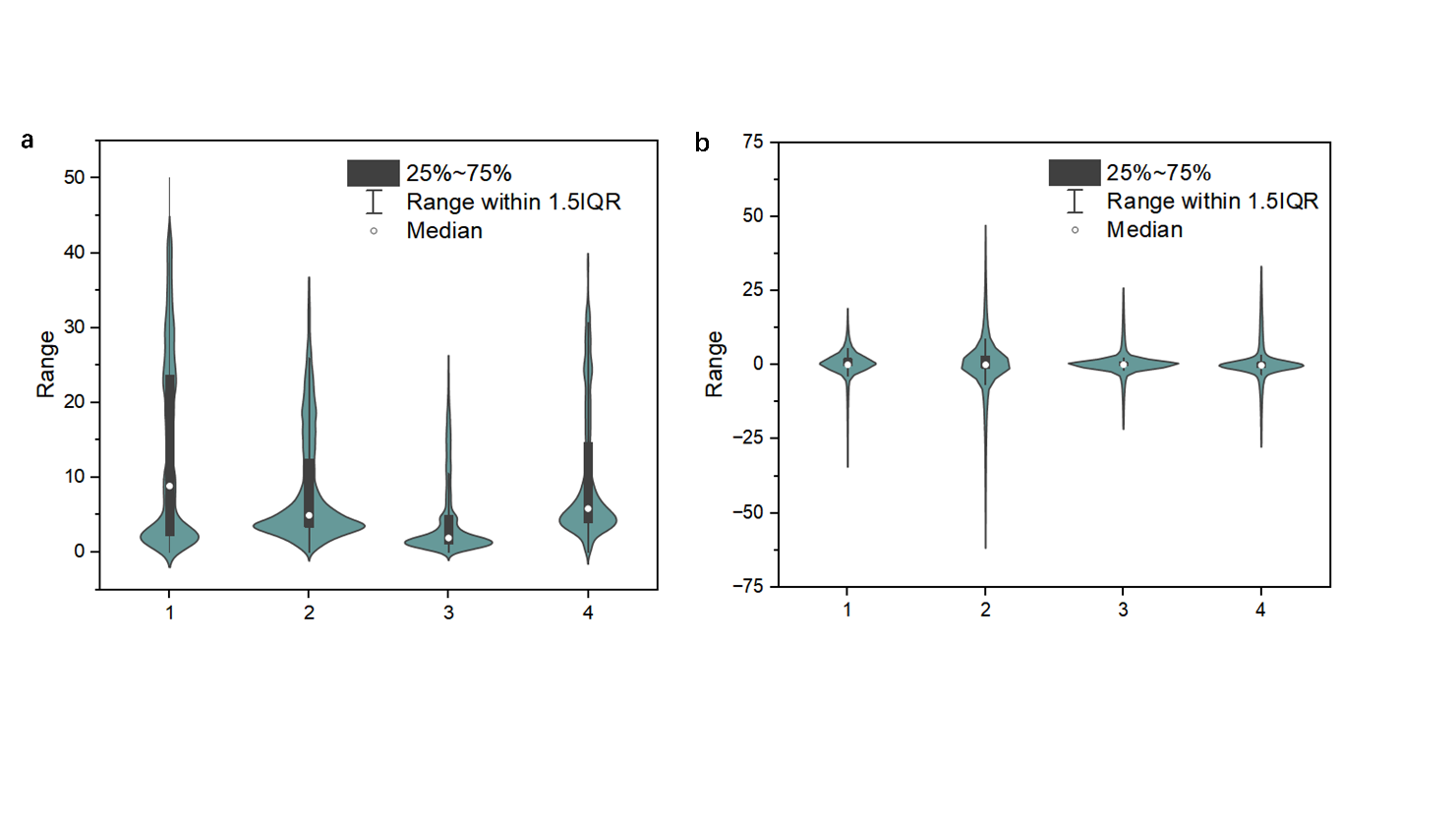


Figure S13. Violin diagram of (a)angle and (b)angular velocity of 4 patients during the experiment.

This graph illustrates the distribution of angular change and angular velocity change across four patients for each data set. The box portion of each violin plot represents the interquartile range (25% to 75%), while the whiskers indicate the minimum and maximum values. The white dots denote the median of each data set. From Figure S10a, patients 1 and 2 exhibit more concentrated data, indicating less variation in angular change, while patients 3 and 4 display a broader range of angular change. Similarly, according to Figure S10b, patients 1 and 2 show a narrower range of angular velocity variation, whereas patients 3 and 4 experience wider fluctuations, suggesting greater angular velocity variability during motion.This graph enables the analysis of differences in angular and angular velocity changes among the four patients, providing insights into their motor characteristics in the motor function rehabilitation experiment.


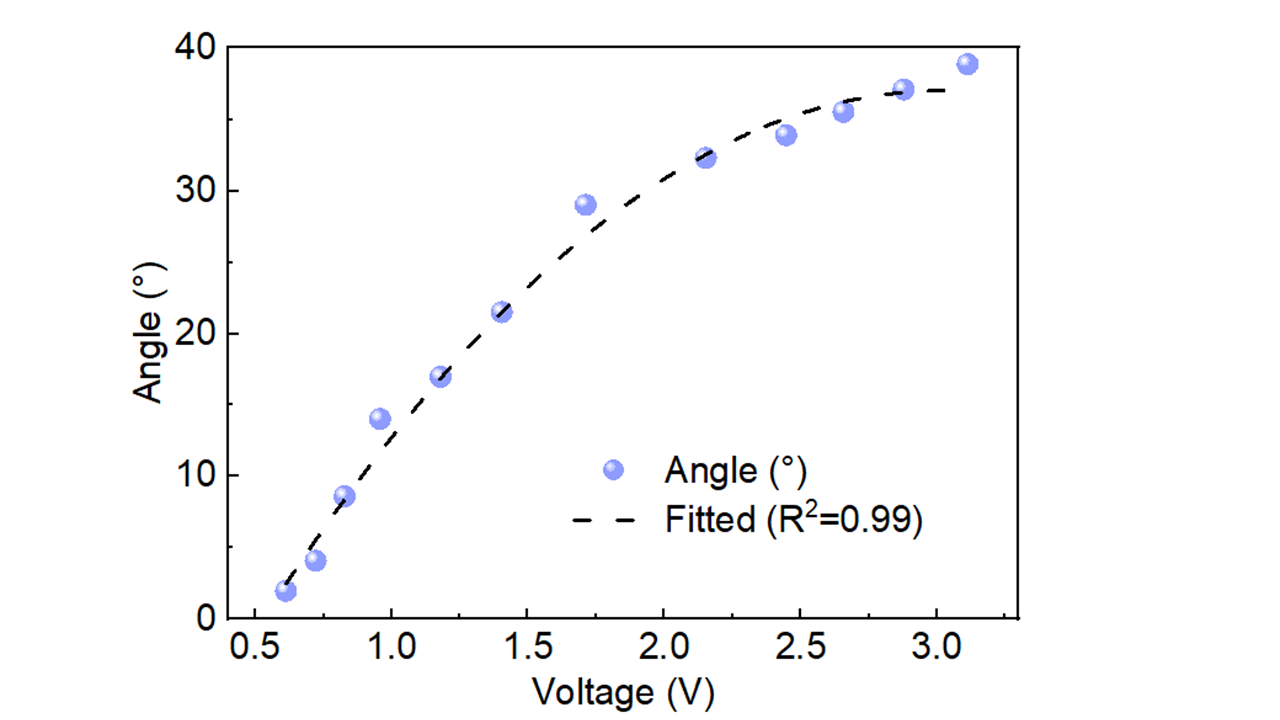


Figure S14. The fitting curve of FMG and angle.

The figure depicts the relationship between the FMG signal and the wrist lift angle, along with the corresponding fitted curve. As the FMG signal increases, the wrist lift angle also rises. However, the rate of angle change diminishes progressively with higher FMG values, indicating that as muscle tension increases, muscle contraction slows down, leading to a more gradual change in wrist lift angle. This trend highlights the nonlinear response of muscle contraction under increasing tension, reflecting the biomechanical characteristics of muscle dynamics.


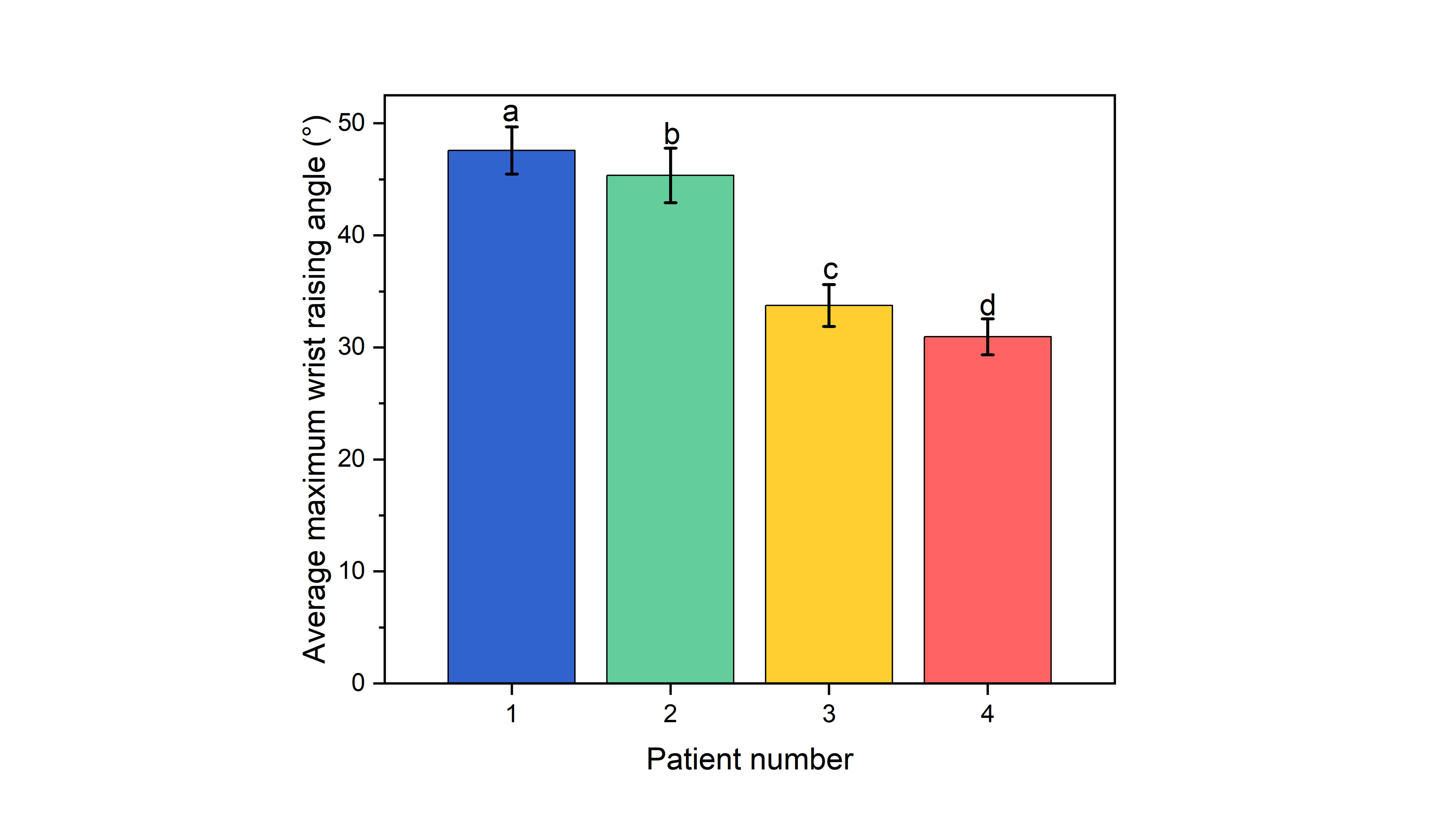


Figure S15. One-way ANOVA analysis of maximum wrist elevation angles. （Different letters indicate significant differences between groups；F(3,56)=250.81, p<0.001, η²=0.93.）

One-way ANOVA analysis of maximum wrist elevation angles across four subjects revealed statistically significant differences (F(3,56) = 250.81, p < 0.001, η² = 0.93). The results demonstrated a clear performance hierarchy: Patient 1 achieved the highest average maximum wrist elevation angle (47.5 ± 2.1°), followed by Patient 2 (45.3 ± 2.4°), Patient 3 (33.7 ± 1.8°), and Patient 4 (30.9 ± 1.6°). Post-hoc Tukey HSD tests confirmed significant pairwise differences among all patients (p < 0.05), as indicated by distinct statistical groupings (a, b, c, d).These substantial individual variations reflect differences in stroke severity, lesion location, time since onset, and baseline motor function. The large effect size (η² = 0.93) underscores the clinical significance of inter-patient variability, highlighting the critical need for personalized rehabilitation protocols tailored to each patient's specific functional capacity and recovery potential.


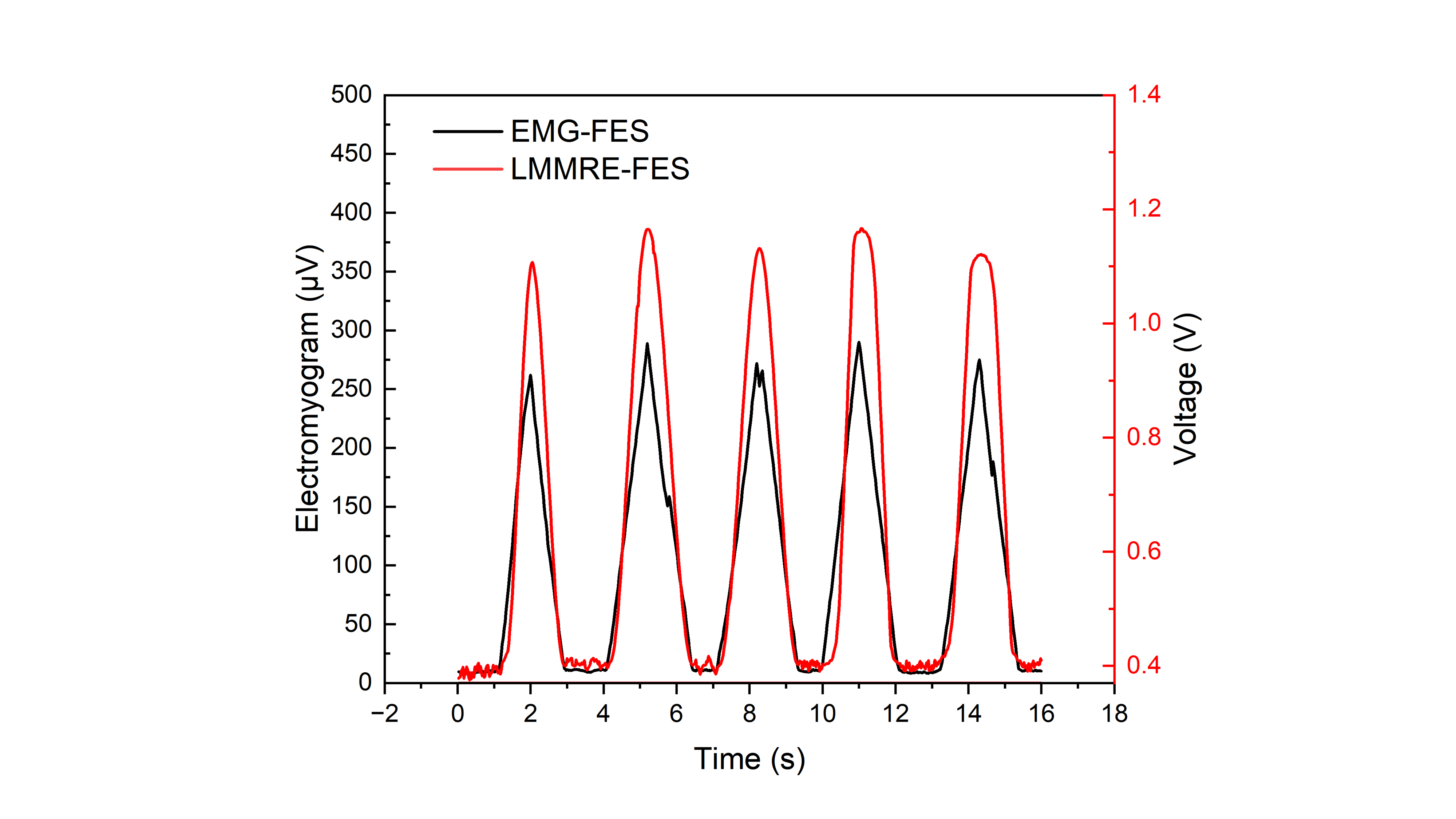


Figure S16. Comparison of signal stability between LMMRE-FES and EMG-driven FES. The figure presents a comparative analysis of voltage signals simultaneously recorded from LMMRE-FES and EMG-driven FES systems during repetitive wrist elevation tasks performed by a single subject equipped with both sensor modalities. Over a 16-second recording period, both systems demonstrated synchronized activation patterns across five consecutive wrist lifting cycles, indicating comparable sensitivity and responsiveness for motor intent recognition.


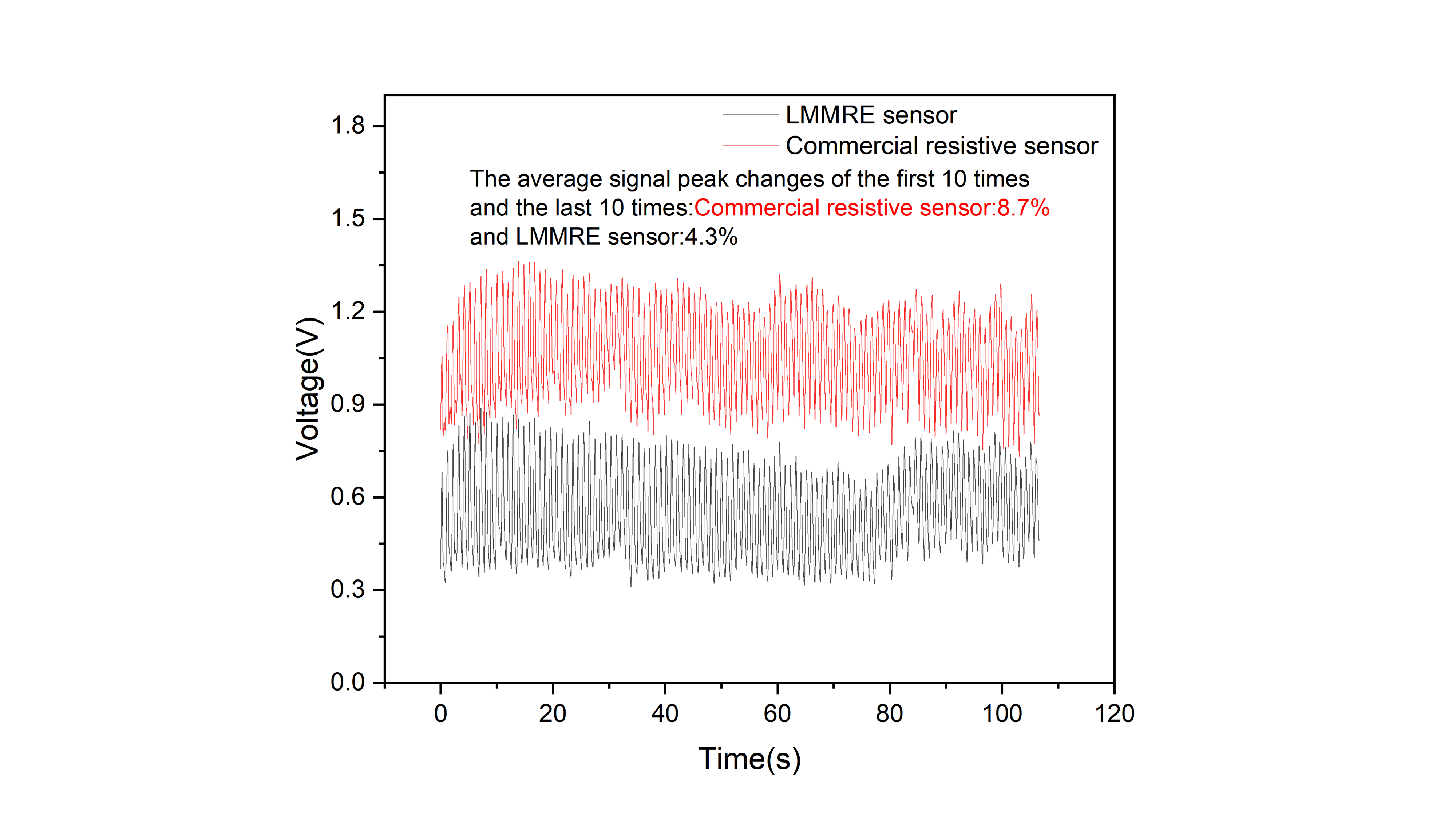


Figure S17. Comparison of skin contact stability.

The figure compares skin-contact stability between the LMMRE sensor and a commercial piezoresistive sensor (IMS-C10A) during 100 consecutive wrist elevation cycles. The LMMRE sensor demonstrated superior stability, with only 4.3% variation in average peak amplitude between the initial and final 10 cycles, compared to 8.7% degradation observed with the commercial sensor.


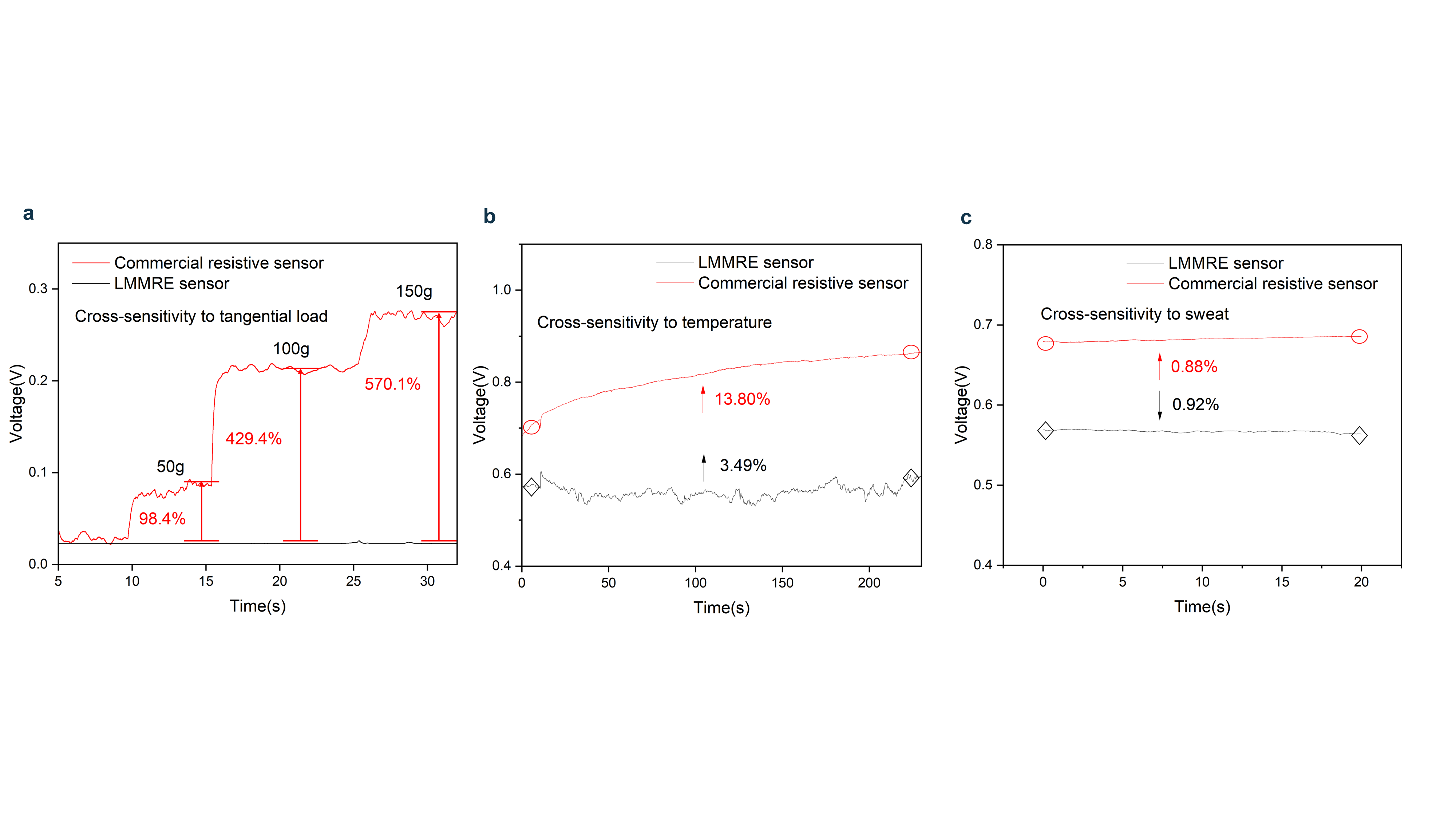


**Figure S18.** **Comparison of Cross-sensitivity. a) Cross-sensitivity to tangential load. b) Cross-sensitivity to temperature. c) Cross-sensitivity to sweat.**

The figure presents a comprehensive cross-sensitivity analysis comparing the LMMRE sensor and commercial piezoresistive sensor (IMS-C10A) under three common interference conditions. **As depicted in Figure S18a,** tangential load assessment demonstrates the LMMRE sensor's superior resistance to lateral force interference, maintaining signal stability with variations below 5% under normal loading conditions, while the commercial sensor exhibited substantial signal fluctuations of 98.4%, 429.4%, and 570.1% under 50g, 100g, and 150g tangential loads, respectively. Temperature cross-sensitivity evaluation (Figure S18b) over 250 seconds reveals remarkable thermal stability of the LMMRE sensor (3.49% signal drift) compared to the commercial sensor (13.80% drift), indicating superior performance consistency across varying environmental temperatures. Sweat interference testing (Figure S18c) shows minimal impact on both sensors when exposed to simulated physiological saline conditions, with comparable performance between the LMMRE sensor (0.92% variation) and commercial sensor (0.88% variation). This multi-dimensional analysis confirms the LMMRE sensor's enhanced robustness against critical clinical interference factors, particularly tangential forces and temperature fluctuations—key considerations for reliable long-term monitoring in rehabilitation applications. The superior interference rejection capability directly translates to improved signal fidelity and reduced false triggering in clinical FES systems.

**Reference**

[S1] S. Stassi, V. Cauda, G. Canavese, C. F. Pirri, Sensors 2014, 14, 5296-5332.

[S2] M. Lenzlinger, E. H. Snow, IEEE Trans. Electron Devices 1968, 15, 686-686.

[S3] B. M. Doucet, A. Lam, L. Griffin, Yale J Biol Med 2012, 85, 201.

[S4] K. Endo, S. Ishigaki, Y. Masamizu, Y. Fujioka, A. Watakabe, T. Yamamori, N. Hatanaka, A. Nambu, H. Okado, M. Katsuno, H. Watanabe, M. Matsuzaki, G. Sobue, Neurosci. Res. 2018, 128, 50-57.

[S5] D. Farina, N. Jiang, H. Rehbaum, A. Holobar, B. Graimann, H. Dietl, IEEE Trans. Neural Syst. Rehabil. Eng. 2014, 22, 797-809.

[S6] A. Belyea, K. Englehart, E. Scheme, IEEE Trans. Biomed. Eng. 2019, 66, 3098-3104.
